# MicroRNA339 Targeting PDXK Improves Motor Dysfunction and Promotes Neurite Growth in the Remote Cortex Subjected to Spinal Cord Transection

**DOI:** 10.3389/fcell.2020.00577

**Published:** 2020-07-21

**Authors:** Liu-Lin Xiong, Yan-Xia Qin, Qiu-Xia Xiao, Yuan Jin, Mohammed Al-Hawwas, Zheng Ma, You-Cui Wang, Visar Belegu, Xin-Fu Zhou, Lu-Lu Xue, Ruo-Lan Du, Jia Liu, Xue Bai, Ting-Hua Wang

**Affiliations:** ^1^Institute of Neurobiological Disease, Department of Anesthesiology, Translational Neuroscience Center, West China Hospital, Sichuan University, Chengdu, China; ^2^Animal Zoology Department, Institute of Neuroscience, Kunming Medical University, Kunming, China; ^3^National Traditional Chinese Medicine Clinical Research Base and Western Medicine Translational Medicine Research Center, Department of Cardiac and Cerebral Diseases, Department of Anesthesiology, Affiliated Traditional Chinese Medicine Hospital, Southwest Medical University, Luzhou, China; ^4^School of Pharmacy and Medical Sciences, Sansom Institute, Division of Health Sciences, University of South Australia, Adelaide, SA, Australia; ^5^Department of Histology and Neurobiology, College of Preclinic and Forensic Medicine, Sichuan University, Chengdu, China; ^6^International Center for Spinal Cord Injury, Kennedy Krieger Institute, Baltimore, MD, United States; ^7^Department of Neurology and Pathology, Johns Hopkins University School of Medicine, Baltimore, MD, United States

**Keywords:** microRNA339, motor cortex plasticity, PDXK, RNA interference, spinal cord injury

## Abstract

Spinal cord injury (SCI) is a fatal disease that can cause severe disability. Cortical reorganization subserved the recovery of spontaneous function after SCI, although the potential molecular mechanism in this remote control is largely unknown. Therefore, using proteomics analysis, RNA interference/overexpression, and CRISPR/Cas9 *in vivo* and *in vitro*, we analyzed how the molecular network functions in neurological improvement, especially in the recovery of motor function after spinal cord transection (SCT) via the remote regulation of cerebral cortex. We discovered that the overexpression of pyridoxal kinase (PDXK) in the motor cortex enhanced neuronal growth and survival and improved locomotor function in the hindlimb. In addition, PDXK was confirmed as a target of miR-339 but not miR-124. MiR-339 knockout (KO) significantly increased the neurite outgrowth and decreased cell apoptosis in cortical neurons. Moreover, miR-339 KO rats exhibited functional recovery indicated by improved Basso, Beattie, and Bresnehan (BBB) score. Furthermore, bioinformatics prediction showed that PDXK was associated with GAP43, a crucial molecule related to neurite growth and functional improvement. The current research therefore confirmed that miR-339 targeting PDXK facilitated neurological recovery in the motor cortex of SCT rats, and the underlying mechanism was associated with regulating GAP43 in the remote cortex of rats subjected to SCT. These findings may uncover a new understanding of remoting cortex control following SCI and provide a new therapeutic strategy for the recovery of SCI in future clinical trials.

## Introduction

Spinal cord injury (SCI) in the shape of hemisection, transection, or crushes cause body movement and sensory and autonomic dysfunction (Courtine et al., 2008; Kao et al., 2009; Yunta et al., 2012; Hu et al., 2013; Chang et al., 2018). As one of the most severe SCI, spinal cord transection (SCT) can result in serious disability and bring about debilities both medically and socioeconomically (Liu et al., 2014; Nas et al., 2015). The evidence showed that SCI usually results in high morbidity of 10–83 cases per million individuals globally (van Middendorp et al., 2011; Ahuja et al., 2017). The consequences of SCI are profound and persistent, eventually leading to short- or long-term neurological dysfunction and disabilities as well as high mortality (Aguilar et al., 2010). Furthermore, existing treatments for SCI have proved scarce, partly due to limited nerve regeneration and an incomplete understanding of post-injury cellular and molecular changes (Li et al., 2008; Furlan et al., 2013; Lee et al., 2014). It is urgent and critically necessary to ameliorate survival and quality of life for individuals with SCI. Previous reports indicated that SCI influences the somatotopic organization of the primary somatosensory cortex, evoking changes in the cortical networks that play a critical role in cortical reorganization after SCI (Kao et al., 2009; Aguilar et al., 2010). Nevertheless, little is known about the key role of the motor cortex in facilitating functional plasticity following SCI. Therefore, investigating the underlying mechanism of the remote cortex in SCI may provide a new strategy to promote the recovery of SCI and potentially contribute to clinical practice.

Pyridoxal kinase (PDXK), a key enzymatic protein, is involved in over 100 enzymatic reactions in the majority of organisms (Huang et al., 2012, 2017). It is a crucial metabolic enzyme of Vitamin B6 that synthesizes a long list of metabolites including pyridoxal (PL), pyridoxine (PN), pyridoxal phosphate (PLP), pyridoxine phosphate (PNP), and pyridoxamine phosphate (PMP) (Kronenberger et al., 2014; Mascolo et al., 2020). PDXK catalyzes the phosphorylation of episomal VitB6 in the presence of Zn^2+^ and ATP (Tang et al., 2005; Safo et al., 2006). Additionally, PDXK converts 4-amino-5-hydroxy-methyl-2-methyl pyrimidine (HMP) into its phosphate ester, HMP-P. Moreover, PDXK and pyridoxine dehydrogenase (PND) determine the synthesis of PLP and regulate the expression level of PLP with phosphatase. PLP, the main active form of VitB6, functions in many biological metabolisms, such as deamination and transamination of amino acids, hemoglobin synthesis, and the decomposition of glycogen (Kerry et al., 1986; Percudani and Peracchi, 2003; Eliot and Kirsch, 2004). Many neurotransmitters are synthesized by PDXK-dependent enzymes, including dopamine, noradrenaline, serotonin, and gamma-amino-butyric acid (GABA) (Dakshinamurti et al., 1990). Researchers agree that PDXK is associated with the pathogenesis of some nervous system diseases (NSDs) such as Down syndrome (Shin et al., 2004; Ramachandran et al., 2015), Parkinson’s disease (Guella et al., 2010; Wider et al., 2010; M’Angale and Staveley, 2017), and seizures (Gachon et al., 2004; Galluzzi et al., 2013). However, the role of PDXK and its binding microRNA in SCI is largely unknown.

MicroRNAs are a category of small non-coding RNA molecules comprising 20–24 nucleotides, playing an essential role in controlling the translation of their target mRNA (Ling et al., 2011; Liu et al., 2013). On the one hand, the complementarity binding between the microRNA and the target gene inhibits the translation without degrading the mRNA, while high levels of complementarity can induce the degradation of mRNA (Han et al., 2013; Liu et al., 2013). The human genome encodes over 1,000 microRNAs. On the other hand, the majority of cellular mRNAs have been evaluated to be controlled by microRNA regulation. Studies have found multiple biological functions of microRNA, such as participating in the regulation of growth and development, proliferation and differentiation, metabolism, inflammatory response, tumors and apoptosis, and many other pathophysiological processes (Ponomarev et al., 2013; Qadir et al., 2013; Chen et al., 2015; Xie et al., 2015). As a result, the abnormal regulation of microRNA expression correlates with various diseases, including SCI (Hu et al., 2013). Recent researches also demonstrated that changes in gene expression following neural injury can be caused by the dysregulation of microRNAs (Yunta et al., 2012; Gaudet et al., 2016; He et al., 2016). In addition, microRNAs play important regulatory roles in spinal cord development, spinal plasticity, and recovery after SCI (Jee et al., 2012; Yunta et al., 2012). The possibility of microRNA targeting PDXK in the remote cortex is largely unknown and needs further investigation.

In our research, applying a two-dimensional differential in-gel electrophoresis (2D-DIGE) and the tandem mass spectrometry (MS/MS) method, we found that the level of PDXK was significantly increased in the motor cortex of rats after SCT. To investigate the function of PDXK and the mechanism associated with its role, we studied the correlation of miR-339 with PDXK in the remote cortex of SCT rats. Our study reported that miR-339 is crucial for PDXK function in the remote cortex subjected to SCT, which therefore may provide a potential strategy for SCT therapy in future clinical trials.

## Materials and Methods

### Animal Care

Adult female Sprague–Dawley (SD) rats weighing 200 ± 20 g provided by the Animal Zoology Department of Kunming Medical University were cared in compliance with the National Institutes of Health’s (NIH) Guide for Care and Use of Laboratory animals. The operations and procedures on rats were approved by the Institutional Animal Care and Use Committee of Kunming University (kmmu2019005). Rats were housed with a 12-/12-h light/dark cycle. Water and food were sufficient. The body temperature of rats was sustained post-SCT operations. Then, bladders of rats were emptied three times a day. A total of 92 SD rats and 9 miR-339 knockout rats were used in this study and randomly arranged into groups, which were detailedly described in Supplementary Tables 1, 2.

### Construction of PDXK Lentivirus ORF/shRNA Expression Plasmid

To detect the effect of PDXK in rats suffering from SCT, a recombinant PDXK open reading frame (ORF) and small hairpin RNA (shRNA) lentiviral vector were designed, constructed by GeneCopoeia^TM^ Company (Guangzhou, China). First, total RNA was isolated from rat’s brain. Then, PDXK-ORF complementary DNA (cDNA) was amplified with 5′-GCGGTAGGCGTGTACGGT-3′, 5′-CTGGAATAGCTCAGAGGC-3′ [designed on the basis of National Center for Biotechnology information (NCBI), accession number: NM_031769]. PDXK DNA fragments were purified by agarose gel, followed by construction of recombinant overexpression plasmid by GeneCopoeia Company.

### Production of Lentiviral Vector

The production procedure was in accordance with the Lenti-Pac^TM^ HIV Expression Packaging Kit User Manual. 293Tα lentiviral packaging cell lines were cultured in Dulbecco’s modified Eagle’s medium (DMEM) with 10% fetal bovine serum (FBS) before reaching a suitable cell confluence for transfection. First, 1.25 μg of the lentivirus expression plasmid (Lenti-Pac^TM^ HIV) was mixed with 2.5 μl (0.5 μg/μl) of the packaging mixture before adding 75 μl of Opti-MEMI^®^ (Invitrogen). Then, in a separate tube, 7.5 μl EndoFectin was mixed with 75 μl Opti-MEM. The mixture was then added dropwise to the carrier mixture in the first mixture and incubated at room temperature for 25 min (min) then added to the 293Tα medium. In order to increase the efficiency of virus production, Titer Boost reagent (10 μl) was added to the cell culture medium, and cells were then incubated for an additional 48 h. The medium with pseudovirus particles was obtained and centrifuged at 4°C for 10 min (2,000 × *g*), which was then mixed with Lenti-Pac concentrated solution at a ratio of 5:1 and incubated for at least 2 h. After that, the medium was collected and centrifuged at 3,500 × *g* for 25 min. The supernatant was carefully discarded; the virus was resuspended through 1/10th of the original volume. Ultimately, the virus was stored at −80°C for further use and study.

### Screening for Knocking Down RNA Fragments

Three potential siRNA sequences and a negative control siRNA were designed to knock down PDXK expression (NCBI accession number: NM_031769.1). Information on the detail is shown in Supplementary Table 3. The three specific pieces of RNA were synthetized by RiboBio Company (Guangzhou, China) and then fused to psiHIV-U6 plasmid. This plasmid was transfected in PC12 cells with transfection reagent (SuperFectin^TM^ II). After 12 h, the basal culture medium was displaced with complete medium consisting of DMEM (Hyclone) with 10% FBS (GIBCO) and 50 U/ml penicillin–streptomycin (Hyclone). Moreover, total RNA was extracted at 48 h post-transfection and further used for cDNA synthesis. T100^TM^ Thermal Cycler (BIO-RAD) PCR amplification was performed, and optical density (OD) readings were applied to compare the products. The most efficient one was then applied to produce recombinant interference lentiviruses as above.

### Lentivirus Transfection in Primary Cortical Neurons

One-day-old pups were anesthetized. After separating the cerebral cortex and gently peeling off of the meninge, the cerebral motor cortex was cut into 1-mm^3^ pieces. Trypsin (0.25%) (Sigma, St. Louis, MO, United States) was used to digest tissue to a cell suspension by gentle pipetting. Next, the cells were resuspended into serum-free medium containing Neurobasal (Life Technologies, Carlsbad, United States), B27 (50×, Life Technologies, Carlsbad, United States), and penicillin/streptomycin (50 U/ml, Hyclone, Utah, United States) and seeded in six-well plate at a density of 1–5 × 10^6^ cell/ml at 37°C. After ∼7 days of culturing, the cells were detected by immunofluorescence staining with Neuronal Class III β-Tubulin (Tuj1) antibody (1:200, Abcam, Cambridge Science Park, England) and then transfected with lentiviral in accordance with the manufacturer’s instructions as described above. To investigate the function of PDXK, primary cultured cortical neurons were randomly arranged into five groups: normal, PDXK ORF-control (ORF-C), PDXK ORF, PDXK shRNA-control (shRNA-C), and PDXK shRNA. Successfully transfected cortical neurons were determined by green and red fluorescence, and pictures were taken at 3 and 5 days after transfection. Five fields of the same area were collected to measure the number and the length of neuritis in cortical neurons via Leica DMI6000B (LAS AF System, Germany).

### Transfection of miR-339 Mimic/Inhibitor or PDXK siRNAs Into Primary Cortical Neurons

To confirm the role of miR-339 on neuronal growth and regulatory relationships with PDXK in function, the transfection of miR-339 mimic/inhibitor and PDXK siRNAs (PDXK-si) was performed. In brief, P1 cortical neurons were cultured for 7 days to reach ∼70% confluence. For the transfection of microRNAs, primary cultured cortical neurons were randomly arranged into nine groups: normal, NC, reagent, mimic-NC, inhibitor-nc, mimic, inhibitor, PDXK-siRNA, inhibitor + PDXK-si. MiR-339 mimic/inhibitor, and PDXK siRNAs were designed and synthesized by RiboBio (Guangzhou, China). Transfection was carried out by adding 3 μl riboFECTTM CP Reagent (Guangzhou, China) to a prepared mix of transfection stock buffer and microRNA or siRNA. The mixture of miR-339 mimic (100 nM), inhibitor (100 nM), or siRNA (100 nM) was added dropwise to the corresponding wells. After incubating in a 1-ml culture system at 37°C for 24 h, 1 ml of new medium was added to system. Cells were detected 4 days after transfection using a fluorescence microscope (Leica CM 1860, Germany). Cell number and area of neurites were counted by Image-Pro Plus 6.0 software (Media Cybernetics, Silver Spring, MD, United States).

### Construction of miR-339 Knockout Rats

MiR-339 knockout rats were constructed using CRISPR-caspase9 gene knockout system by Cyagen Biosciences Inc. (Guangzhou, China^1^). After F1 generation was acquired, they were divided into wild type (WT), heterozygotes, and homozygotes. Then, they were mated to procure more homozygotes. Genotypes of the newborn rats were detected by PCR within 24 h after birth. For the purpose of further confirming the function of miR-339 and its relationships with PDXK, neonatal rats were used to culture cortical neurons and grouped according to genotypes. In addition, the adult female miR-339 knockout rats were subjected to SCT, and Basso, Beattie, and Bresnehan (BBB) score was performed at 3, 7, 14, and 28 days post-operation to observe the functional recovery.

### SCT Surgery and Lentivirus Injection

After construction and identification of PDXK Lentivirus overexpression/interference (ORF/shRNA) plasmid, they were used to perform the following experiments (Supplementary Figure 1). The details about the PDXK siRNA are shown in Supplementary Table 3. Surgical procedures were performed as formerly described (Liu et al., 2015; Wang et al., 2016). In brief, using a glass micropipette, 10 μl Lentivirus (0.2 μl/min) was injected into the cerebral cortex motor area into four spots, 2.5 μl for each point. The empty vector was also injected to be used as negative control.

In the procedure of establishment of SCT, rats were anesthetized with 2% sodium pentobarbital sodium (30 mg/kg). Rats underwent laminectomy of T9–11 to expose 1.5-cm long of the spinal cord. Then, spinal cord was completely transected at T10, and the intervening tissue was removed. To make certain the completeness of the transection, the cut ends were lifted with small forceps. Animals in the sham operation group were subjected to the same procedure excluding T10 transection.

### Basso, Beattie, and Bresnehan Scores

The BBB locomotor rating scale was used as the criteria for functional recovery in SCI model rats and implemented as formerly reported (He et al., 2016). The BBB scores grade from 0 point (no visible hindlimb movement) to 21 points (normal mobility). In brief, assessments were performed at 3, 7, 14, and 28 days post-operation (dpo) in an open field by three investigators who were not aware of the experiment design. Rats were put into an open field for 3 days prior to detection for 5 min/day. After SCT surgery, three experienced investigators evaluated each animal for 4 min and assigned an operative defined score for each hindlimb. The mean score of investigators was served as the final score for analysis.

### Tissues Harvesting

After anesthesia with 3.6% chloral hydrate (50 mg/kg, intraperitoneally), femoral artery was disengaged; then, motor areas of the cerebral cortex, heart, liver, spleen, kidney, lung, adrenal glands, hypophysis, hippocampus, cerebellum, and spinal cord were separated, respectively. Samples were rapidly stored at −80°C before analysis. For immunofluorescent staining, animals were deeply anesthetized; then, intracardiac perfusion was conducted for 5 min using isotonic saline and sequentially for 5 min using 4% par formaldehyde. After the perfusion, cortexes were removed for Feminizing Locus on X-3, Fox-3, Rbfox3, or hexaribonucleotide binding protein-3 (NeuN), PDXK, and terminal deoxyribonucleotidyl transferase-mediated dUTP-digoxigenin nick-end labeling (TUNEL) staining.

### Proteomics Mass Spectrometry Analysis

Cortex samples from sham and SCT groups were stained with 0.1 ml of 50 mM NH_4_HCO_3_ and 50% acetonitrile for 20 min, then dehydrated in 100% acetonitrile for 10 min, and then, the spots were incubated at 37°C for 5 min. Ten microliters trypsin (12.5 mg/ml) were added at 4°C for 30 min followed by overnight incubation at 37°C. Next, peptides were extracted twice by 0.1% trifluoroacetic acid (TFA) and 50% acetonitrile for 30 min, then dried with nitrogen stream, and then dissolved in 0.8 μl of mixed liquid α-cyano-4-hydroxycinnamic acid (α-CHCA, Sigma, St. Louis, MO, United States), 0.1% TFA, and 50% acetonitrile. Results were calculated by a 4700 Proteomics Analyzer (Applied Biosystems, Foster City, CA, United States). Using the internal calibration mode, the trypsin-digested myoglobin peptides were added to six calibration spots on the matrix-assisted laser desorption/ionization (MALDI) plate to calibrate the mass equipment. A 20-kV accelerating voltage was performed to the sample plate, and then, an ultraviolet (UV) laser was operated at a repetition frequency of 200 Hz with a wavelength of 355 nm. In default mode, data interpretation of the gained mass spectrometry (MS) and MS/MS peptide spectra were performed using Data Explorer TM software (version 4.5, Applied Biosystems, Foster City, CA, United States). Parent mass peak was chosen for tandem time-of-flight (TOF/TOF) analysis. The generated MS and MS/MS spectra were then transmitted to MASCOT (version 2.1, Matrix Science, London, United Kingdom) via GPS Explorer software (version 3.6, Applied Biosystems, Foster City, CA, United States).

The following are the criteria for identification of proteins: database, NCBI; retrieval mode, combined; mass range of error, PMF 0.3 Da, MS/MS 0.4 Da; species, *Rattusnorvegicus*; enzyme, trypsin; and maximum missed cleavages, 1. Of the five largest hits reported in the SwissProt database, the highest Mowse score (at least 25% coverage) with unique peptide has been verified by Western blot and can be applied to debride the excessiveness of matching protein to numerous members of the protein family (Shang et al., 2013; Liu et al., 2015).

### Quantitative RT-PCR

After the brain cortex from each group was harvested, total RNA was isolated from fresh or briefly frozen samples via Trizol method and reversely transcribed into cDNA. The primer sequences of genes were used as follows: PDXK, 5′GCGGTAGGCGTGTACGGT3′ (forward); 5′CTGGAATAGCTCAGAGGC3′ (reverse); and housekeeping gene β-actin, GAAGATCAAGATCATTGCTCCT (forward); TACTCCTGCTTGCTGATCCA (reverse). MiR-124: miRQ0000828-1-1 Bulge-Looprno-miR-124 quantitative reverse transcription PCR (qRT-PCR) Primer Set (RiboBio, Guangzhou, China) and MiR-339: miRQ0000583-1-1 Bulge-Looprno-miR-339 qRT-PCR Primer Set (RiboBio, Guangzhou, China) were used. The conditions for PCR amplification were as follows: 2 min initial denaturation at 95°C, 15 s denaturation at 95°C and amplification at 53°C (β-actin) and 52°C (PDXK) for 20 and 30 s extension at 60°C, which lasted for a grand total of 40 cycles. The critical threshold (Ct) of each specimen was collected, and all data were normalized to β-actin values by the 2^–△^^△^^*Ct*^ method (Livak and Schmittgen, 2001).

### Western Blot

The Western blot experiment was carried out as previously described (Liu et al., 2014). In brief, tissues from the brain cortex were dissociated and homogenized by radioimmunoprecipitation assay (RIPA) lysis buffer containing protease inhibitor cocktail (Beyotime, Jiangsu, China). A volume of about 80 μg proteins was resolved in 15% sodium dodecyl sulfate (SDS)-polyacrylamide gel (Beijing Junyi Electrophoresis Co., Ltd., Beijing, China) with electrophoresis buffer at 60 V for 30 min, then 100 V for 90 min. The proteins bands were transferred to a polyvinylidenedifluoride (PVDF) membrane with 300 mA for 40 min. For blocking, the membrane was incubated in 5% skim milk at room temperature for 1 h and then was incubated with the primary antibody of PDXK (mouse, Abcam, 1:2,000) and β-actin (mouse, Abcam, 1:5,000) at 4°C overnight. The next day, the membrane was rinsed with 1 × TBS containing 0.2% Tween-20 (TBST; Shanghai Double-Helix Biotech Co., Ltd., Shanghai, China) for four times. Then, the membrane was incubated with 1:5,000 secondary antibody (goat antimouse IgG, ZSGB-BIO, China). Finally, the membrane was rinsed in TBST for four times, and the immune complexes were developed with enhanced chemiluminescence reagent and visualized by ChemiDoc XRS System with Image Lab Software 2.0 (Bio-Rad Laboratories, Inc., Hercules, CA, United States).

### Immunofluorescent Staining

The brain cortex was isolated and soaked in a 4% paraformaldehyde solution for 24 h and then incubated in a 20% sucrose phosphate buffer at 48°C overnight. The specimens were frozen in liquid nitrogen and sectioned serially at 20-μm thickness. Slices were taken out and rinsed with phosphate buffered saline (PBS) for four times (5 min for each time). For blockage, sections were incubated with 10% *v*/*v* normal goat serum PBS-Triton 0.3% *v*/*v* (PBST) at room temperature for 60 min. Then, slices were incubated with PBST containing 5% goat serum and 1:100 anti-PDXK antibody (mouse, Abcam, Cambridge Science Park, England) and 1:50 anti-NeuN antibody for neuron (rabbit, ZSGB-BIO) overnight at 4°C. After that, the slices were washed four times with PBS. Slices were incubated with secondary antibody, Alexa594 (goat antimouse, Abcam, Cambridge Science Park, England, 1:500) and Alexa488 (goat anti-rabbit, Abcam, Cambridge Science Park, England, 1:500), respectively, for 2 h at 37°C. After four washings (5 min for each time) in PBS, 4,6-diamidino-2-phenylindole (DAPI) was performed to mark the nucleus. Lastly, images were recorded by Leica DMI6000B (LAS AF system, Germany). In the side of quantity analysis, a total of five fields of the same area in each group were randomly collected at 400×. The percentage of NeuN^+^ PDXK^+^/DAPI was counted by Image Pro Plus 6.0 software.

### Terminal Deoxyribonucleotidyl Transferase-Mediated dUTP-Digoxigenin Nick-End Labeling Staining *in vitro* and *in vivo*

TUNEL staining was applied *in vivo* to detect cell apoptosis in the cortex of the rats. First, TUNEL staining was performed *in vivo*. In brief, slices were kept in 5% goat serum containing 0.03% Triton-100 for 30 min at 37°C, followed by three times wash with PBS. Afterward, slices were incubated with PBST containing 2% goat serum and 1:50 anti-NeuN antibody (rabbit, ZSGB-BIO) at 4°C for 18 h, and then rinsed with PBS. Subsequently, slices were incubated with secondary antibody, Alexa488 (goat anti-rabbit, Abcam, Cambridge Science Park, England, 1:500), and TUNEL Detection Kit (Roche Molecular Biochemicals, Mannheim, Germany), respectively, for 2 h at room temperature. Lastly, the slices were washed with PBS and mounted with DAPI. Five fields were obtained by Leica DMI6000B (LAS AF system, Germany). Double NeuN and TUNEL-positive cells were related to the NeuN-positive cells to calculate the percentage of the apoptotic cells with the help of Image-Pro Plus 6.0 software (Media Cybernetics, Rockville, MD, United States).

Similarly, apoptosis was determined by TUNEL staining *in vitro*. The cortical neurons were fixed with 4% paraformaldehyde for 15 min, and then, cortical neurons were kept in 0.1% sodium citrate containing 0.03% Triton-100 for 30 min at 37°C, followed by three times wash with PBS. After that, neurons were incubated with PBST containing 2% goat serum and 1:200 anti-Tuj1 antibody (rabbit, Abcam, Cambridge Science Park, England) at 4°C for 18 h, followed by three times wash with PBS. Neurons were incubated with secondary antibody, Alexa488 (goat anti-rabbit, Abcam, Cambridge Science Park, England, 1:500), and TUNEL Detection Kit (Roche Molecular Biochemicals, Mannheim, Germany), respectively, for 2 h at room temperature. Finally, nucleuses were counterstained with DAPI and visualized by fluorescence microscopy (Leica, CM1860, Germany). Random five fields were recorded for each well; cells in the field were counted through a researcher who is unaware of the label of the samples. Double Tuj1- and TUNEL-positive cells were related to the Tuj1-positive cells to calculate the percentage of the apoptotic cells with the help of Image-Pro Plus 6.0 software (Media Cybernetics, Rockville, MD, United States).

### Bioinformatics Prediction

The prediction database TargetScan^2^, miRDB^3^, miRanda^4^, and miRwalk^5^ were used for predicting microRNAs that regulated the target gene, PDXK. The intersecting parts of retrieval results from these databases were selected using venny2.1^6^. In addition, GeneMANIA^7^ was also used to predict the possible relationship with GAP43.

### Luciferase Reporter Assay

Luciferase reporter assay was applied to investigate the relationship between miR-124, miR-339, and PDXK 3′ untranslated region (UTR) wild type (WT), PDXK 3′UTR-Mutant (Mut)-2, PDXK 3′UTR -Mut-3, and PDXK 3′UTR-Mut-4. The assay was determined according to the method previously reported (Zhang et al., 2014). After building the vectors of PDXK 3′UTR-WT, PDXK 3′UTR-Mut-2, PDXK 3′UTR-Mut-3, and PDXK 3′UTR-Mut-4 by RiboBio (Guangzhou, China) as described in Supplementary Table 4, they were cotransfected with miR-124 and miR-339 in 293T cells. For the transfection, cells were seeded into 96-well plates with 1.5 × 10^4^/well with a total 100 μl medium and incubated in 37°C for 24 h. Then, vectors and 0.25 μl Lipofectamine TM 2000 reagent (supplied by RiboBio, Guangzhou, China) were diluted using 10, 15, and 25 μl OPTI-MEM medium and kept in room temperature for 20 min. The transfection mixtures were then added to wells. The concentration of mimics was 50 nM, and the plasmid was 100 ng/ml, and each group had three repeated wells. After 6 h, 100 μl fresh medium was added to each well. Forty-eight hours post-transfection, luciferase activity was measured by a Dual-Luciferase Reporter Assay Kit (Promega, E1910, Madison, Wisconsin, United States). In brief, the medium was sucked out, and 35 μl/well new medium and 35 μl/well luciferase substrate were added. The plate was vibrated for 10 min before and after adding 30 μl stopping reagent. Finally, fluorescence values were detected using a fluorescence light meter.

### Statistical Analysis

The experimental data were in the form of the mean ± standard deviation (SD). The difference between the two groups is compared using a Student’s *t* test with a two-tailed distribution, and multiple group comparisons were performed by one-way analysis of variance (ANOVA) via SPSS 17.0 (SPSS, Inc., Chicago, IL, United States). *P* < 0.05 displays that the difference is statistically remarkable. *P* < 0.05 is displayed by ^∗^; *P* < 0.01 is represented by ^∗∗^; and *P* < 0.001 is indicated by ^∗∗∗^.

## Results

### Verification of Differential Expression of Proteins in Cortex of Rats After SCT at Different Times

**FIGURE 1:**
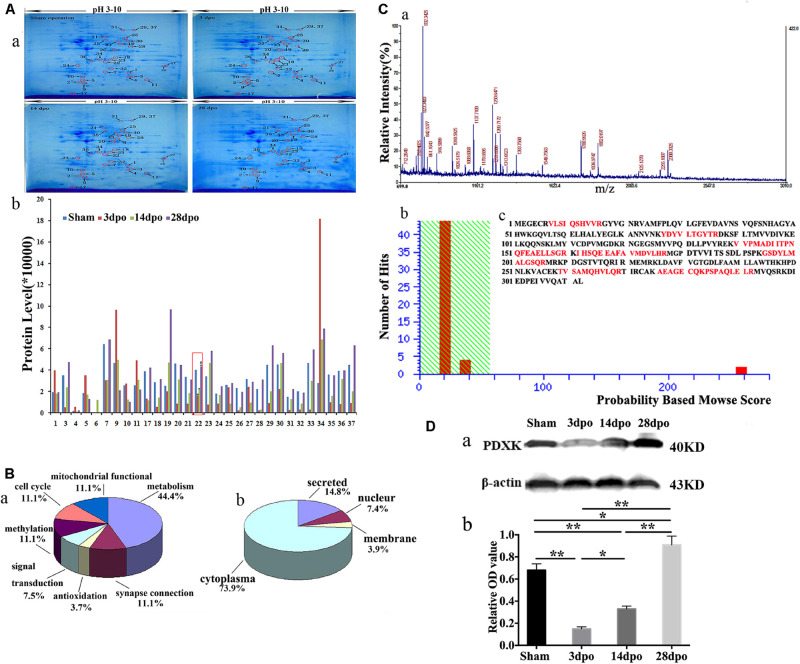
Proteomic analysis in the cortex of spinal cord transection (SCT) rats and matrix-assisted laser desorption ionization-time of flight mass spectrometry (MALDI-TOF-MS) spectrum as well as the verification of PDXK. **(A-a)** Representative 2-DE maps of cortex in the sham, 3, 14, and 28 dpo groups were displayed in polyacrylamide gel with CBB G-250 staining. **(A-b)** Protein quantitative analysis for 30 differential spots.* > 1.5-fold change vs. sham group; ^#^ > 1.5-fold change vs. 3 dpo group. **(B-a,b)** Classification of proteins based on their functions and subcellular localization. **(C-a)** Primary mass spectrometry result of spot 22 (PDXK). **(C-b)** The results of probability-based Mowse score showed that the differential expression of protein is PDXK, where Mowse score is 258 (>56, *P* < 0.05). **(C-c)** The amino acid sequence of PDXK and matching peptides was exhibited in bold red with 31% of matching percentage. **(D-a,b)** The expression level of PDXK was tested by Western blot and normalized to β-actin by Image J. Data were presented as mean ± SD. *P < 0.05; **P < 0.01. dpo, days post-operation; CBB, coomassie brilliant blue, PDXK, pyridoxal kinase.

The 2-DE patterns of sham, 3-, 140, and 28-dpo groups after SCT were exhibited in polyacrylamide gel with Coomassie Brilliant Blue (CBB) G-250 staining (Figure 1A-a). Using PDQuest software, it was found that there were ∼550 protein spots in the sham group, and 412, 351, and 357 protein spots in the 3, 14, and 28 dpo groups, respectively. Among the differentially expressed proteins, 30 spots were expressed at least 1.5-fold as shown by MALDI-time of flight mass spectrometry (MALDI-TOF-MS) quantification (Figure 1A-b). Thirty-seven protein spots were detected by mass spectrum analysis, and the detailed data of these spots are displayed in Supplementary Table 5. Then, these proteins were classified into different groups based on their functions and subcellular localization (Figure 1B), in which, 3 of them were involved in mitochondrial function, 3 in cell cycle, 3 in methylation, 2 in signal transduction, 12 in metabolism, 1 in antioxidation, and 3 in synapse connection (Figure 1B-a), while 20 proteins were located in the cytoplasm, 2 in the nucleus, 1 in the membrane, and four proteins were secreted proteins (Figure 1B-b). Myoglobin-promoting peptide was used as an external calibrator; peptide mass fingerprint (PMF) and MS/MS analysis were carried out, and then verified by GPS-MASCOT for database search (Figure 1C-a). Among them, PDXK was downregulated at 3 days, then upregulated at 14, 28 days in the cortex of rats after SCT. By means of the database search, PDXK (gi| 13929082) gained a score of 258 (score more than 56 will be considered) (Figure 1C-b), additional information about the mass spectrometry data are shown in Supplementary Mass Spectrum Data 1, Supplementary Tables 6, 7. The amino acid sequence matched percentage was 31%, and red colored amino acids reflect the same amino acids (Figure 1C-c). Moreover, in comparison of the sham group, the expression of PDXK was reduced significantly at 3 dpo, then remarkably upregulated at 14 and 28 dpo in the SCT rats (Figure 1D-a,b).

### Function of PDXK on the Neurite Growth of Cortical Neurons *in vitro*

**FIGURE 2:**
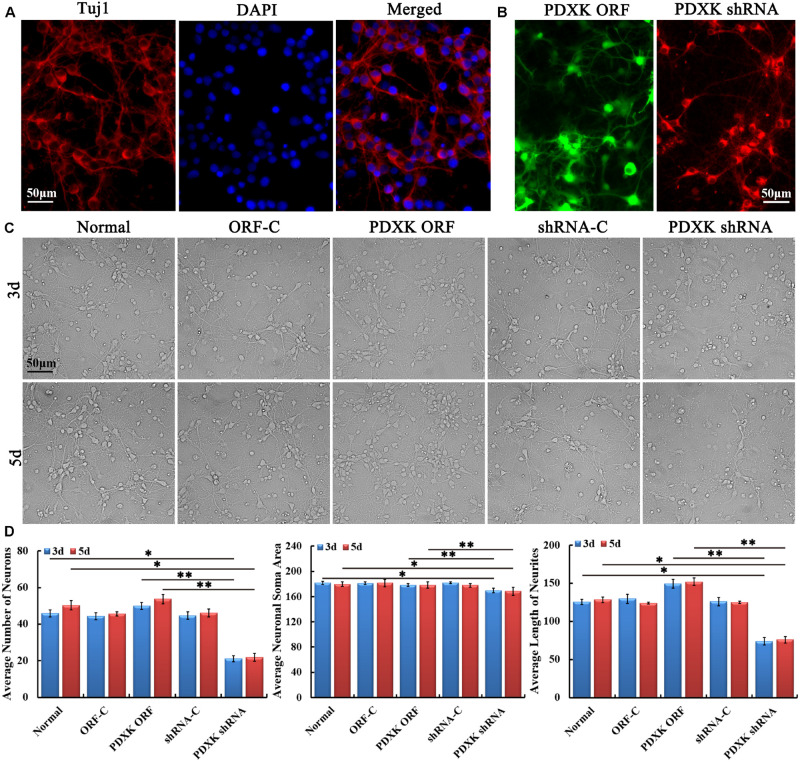
Growth characteristics of primary cultured cortical neurons in rats after transfecting with PDXK-ORF/shRNA Lentivirus. **(A)** Tuj1 staining for cortical neurons at 7 days after culturing. Scale bar = 50 μm. **(B)** PDXK ORF virus production carrying eGFP and PDXK shRNA virus production with mcherryFP were transfected into primary cortical neurons, respectively. Scale bar = 50 μm. **(C)** Primary cultured cortical neurons were transfected with Lentivirus for 3 and 5 days. Scale bar = 50 μm. **(D)** The number, soma size, and neurites’ length of cortical neurons were measured using Leica AF6000 system and quantified after transfected with Lentivirus for 3 and 5 days. Data were shown as mean ± SD. *P < 0.05, **P < 0.01. eGFP, enhanced green fluorescent protein; PDXK, pyridoxal kinase; C, control.

Firstly, primary cultured cortical neurons from neonatal rats on day 7 were identified by Tuj1 staining (Figure 2A). Then, primary cortical neurons were transfected with PDXK ORF-C, PDXK ORF, PDXK shRNA-C, and PDXK shRNA, respectively (the detailed information are described in Supplementary Figure 1). Three days after transfection, the eGFP and mcherryFP fluorescence were visualized under a fluorescent microscope (Figure 2B). Then, the morphology of cortical neurons was captured at 3 and 5 days after transfection (Figure 2C). Compared with the normal group, the number, neurite length, and soma size of cortical neurons were obviously decreased in PDXK shRNA group at 3 and 5 days post-transfection (^∗^*P* < 0.05), while the number, neurite length, and soma size of cortical neurons were markedly increased in PDXK-ORF group at 3 and 5 days post-transfection in comparison of PDXK shRNA group (^∗∗^*P* < 0.01) (Figure 2D).

### PDXK Overexpression in Cortex Promoted Motor Function Recovery and Neuron Survival and Decreased Neuronal Apoptosis

**FIGURE 3:**
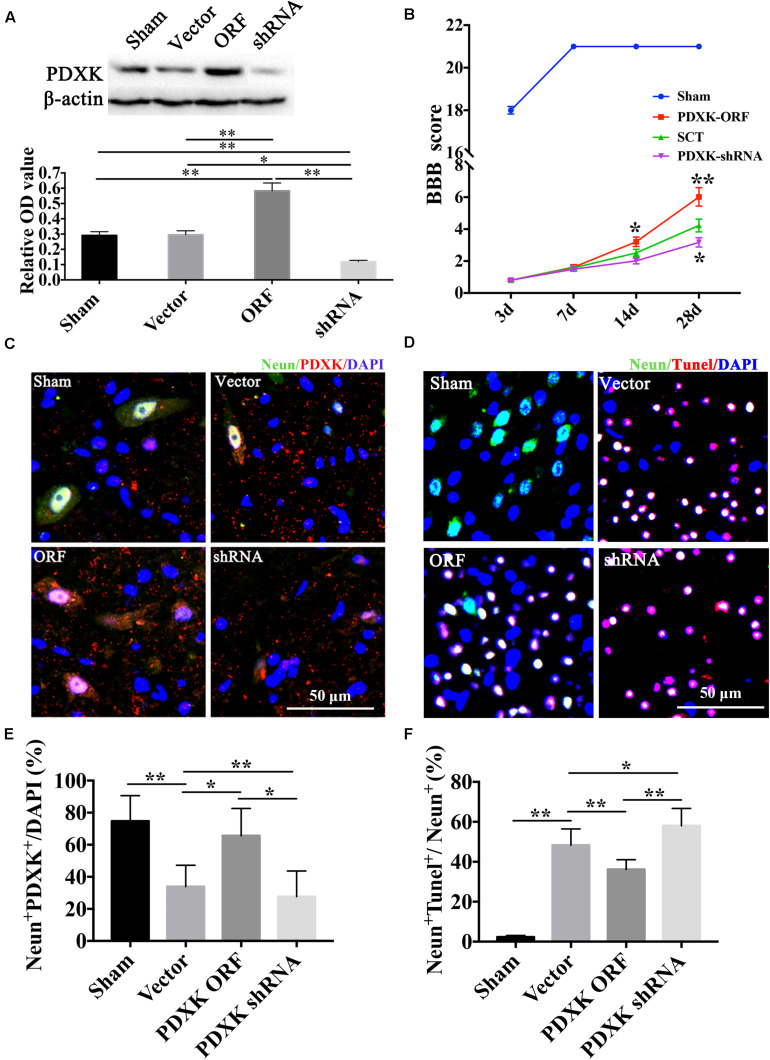
BBB score, NeuN immunofluorescent staining, and TUNEL assay of the cerebral cortex motor area in Lentivirus-treated rats. **(A)** The expression of PDXK following PDXK ORF and PDXK shRNA transfection in the cortex of SCT rats by Western blot. **(B)** The locomotor function was assessed by BBB score. **(C)** Pictures of neurons collected by double immunofluorescent staining of NeuN and PDXK in the cerebral cortex motor area. **(D)** Quantitative column diagram of number of NeuN and PDXK double-positive cells was obtained by Leica AF6000 system. Scale bar = 50 μm. *P < 0.05; **P < 0.01. **(E)** Pictures of apoptotic cells measured by NeuN immunofluorescent staining and TUNEL staining, presented by NeuN + Tunel + /NeuN + (%) in the cerebral cortex motor by Leica AF6000 system. **(F)** Quantitative histogram of neuronal apoptosis. Scale bar = 50 μm. *P < 0.05; **P < 0.01. Data were exhibited as mean ± SD. BBB, Basso, Beattie, and Bresnehan; TUNEL, terminal deoxyribonucleotidyl transferase-mediated dUTP-digoxigenin nick-end labeling; PDXK, pyridoxal kinase; SCT, spinal cord transection; DAPI, 4, 6-diamidino-2-phenylindole.

The expression of PDXK was confirmed by Western blot after PDXK ORF/shRNA Lentivirus injection into the cortex of SCT rats (Figure 3A). As a result, the expression of PDXK was significantly increased in the PDXK ORF group and strikingly reduced in the PDXK shRNA group when compared with sham group (^∗∗^*P* < 0.01). In addition, behavior test showed that PDXK ORF significantly increased BBB score at 14 and 28 day (^∗^*P* < 0.05; ^∗∗^*P* < 0.01), while BBB score was markedly reduced in the PDXK shRNA group at 28 day when compared with the SCT group (^∗^*P* < 0.05) (Figure 3B). The analysis of NeuN^+^PDXK^+^ and TUNEL-positive neurons were performed in the cerebral cortex motor areas in each group. SCT rats treated with Lv-PDXK ORF exhibited a significant increase in the percentage of NeuN^+^PDXK^+^ neurons and a less TUNEL-positive neurons staining around the cortical areas injured in comparison of those treated with Lv-Vector (^∗^*P* < 0.05; ^∗∗^*P* < 0.01), whereas reverse results were seen in rats injected with Lv-PDXK shRNA (Figures 3C–F).

### MiR-339 but Not miR-124 Could Perfectly Regulate the Position of 3′ UTR in PDXK to Prevent the Translation of PDXK

**FIGURE 4:**
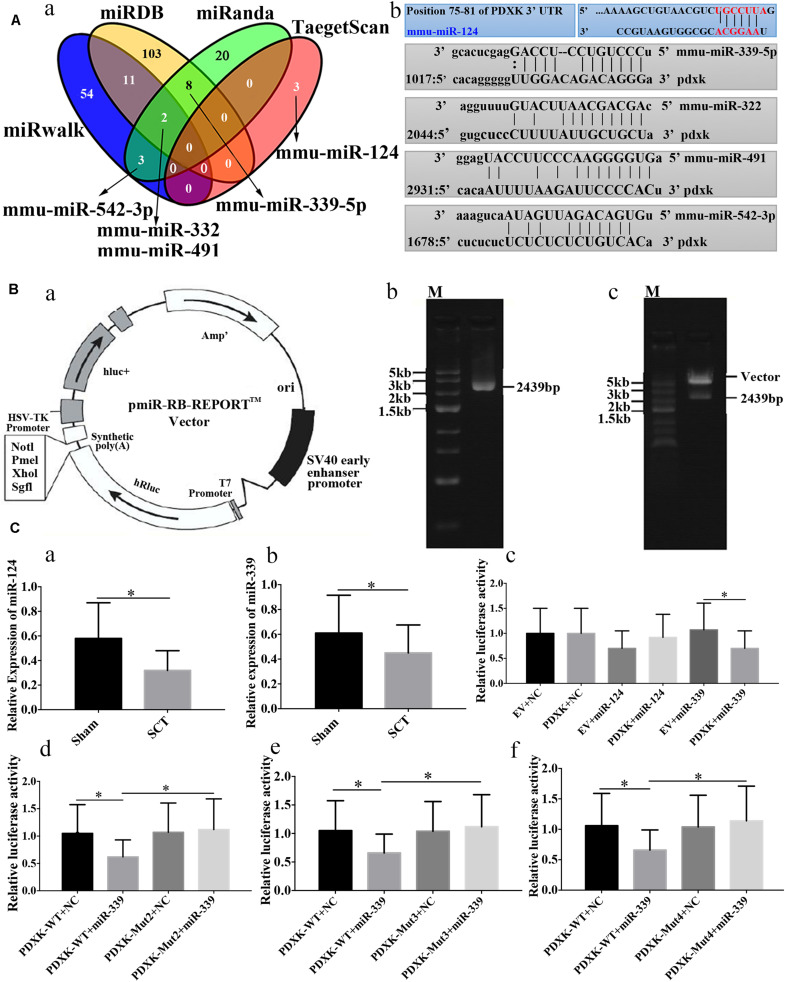
Bioinformatic prediction on PDXK regulated by miRNAs and the validation of their relation. **(A-a)** The map of bioinformatics prediction through TargetScan (http://www.targetscan.org/vert_72/docs/help.html), miRDB (http://www.mirdb.org), miRanda (www.microrna.org), and miRwalk (http://mirwalk.umm.uni-heidelberg.de) databases; venny2.1 (http://bioinfogp.cnb.csic.es/tools/venny/index.html) was implemented to acquire the intersecting results of these four databases. Mmu-miR-124, mmu-miR-322, mmu-miR-491, mmu-miR-339-5p and mmu-miR-542-3p were selected as the candidate miRNAs. **(A-b)** The alignments of predicted microRNAs and PDXK. **(B-a)** Vector information for the vector of PDXK 3′UTR. **(B-b)** One percent agarose electrophoresis analysis of PCR products that amplified PDXK 3′UTR. M, marker5000. **(B-c)** Validation of plasmid enzyme digestion. M, marker5000. **(C-a)** Verification of miR-124 expression in cerebral cortex of rats with SCT at 28 days. **(C-b)** Verification of miR-339 expression in cerebral cortex of rats with SCT at 28 days. **(C-c)** The luciferase assay results in EV + NC, PDXK + NC, EV + miR-124, PDXK + miR-124, EV + miR-339, and PDXK + miR-339 group. **(C-d)** The luciferase assay results in PDXK-WT + NC, PDXK-WT + miR-339, PDXK-Mut2 + NC, and PDXK-Mut2 + miR-339 group. **(C-e)** The luciferase assay results in PDXK-WT + NC, PDXK-WT + miR-339, PDXK-Mut3 + NC, and PDXK-Mut3 + miR-339 group. **(C-f)** The luciferase assay results in PDXK-WT + NC, PDXK-WT + miR-339, PDXK-Mut4 + NC, and PDXK-Mut4 + miR-339 group. Data were exhibited as mean ± SD; *P < 0.05. PDXK, pyridoxal kinase; SCT, spinal cord transection; EV, empty vector; NC, negative control; WT, wild type.

Prediction of microRNAs targeting PDXK has revealed that 54, 103, 20, and 3 target sites exist in the region of PDXK mRNA at 3′UTR as per “miRwalk,” “miRDB,” “miRanda,” and “TargetScan,” respectively (Figure 4A-a). In addition, mmu-miR-124, mmu-miR-322, mmu-miR-491, mmu-miR-339, and mmu-miR-542-3p were predicted as miRNA sequences targeting the PDXK mRNA according to preliminary analysis. The alignments of these microRNAs and PDXK are shown in Figure 4A-b. To illustrate the interaction between miR-124 or miR-339 and PDXK mRNA, PDXK 3′UTR segment was cloned, which includes a possible target site for miR-124 and miR-339, while the downstream of the hRluc luciferase reporter gene was inserted to produce the hRluc-PDXK vector (Figure 4B-a). The identification of hRluc-PDXK vector and the PCR product amplified by PDXK 3′UTR are shown in Figures 4B-b,c and Supplementary Figure 2. The qRT-PCR was performed to determine miR-124 and miR-339 expression in cerebral cortex of rats with SCT, and the results demonstrated that miR-124 and miR-339 expression were significantly decreased in cerebral cortex of rats at 28 days post-SCT (^∗^*P* < 0.05) (Figures 4C-a,b). The results of luciferase analysis exhibited that miR-339, but not miR-124, can decrease markedly luciferase activity, compared with NC and empty vector (^∗^*P* < 0.05) (Figure 4C-c). In order to test whether the miR-339 binding sites located in the PDXK 3′UTR (WT) is responsible for the microRNA-mediated suppression of target gene expression, the point mutations were introduced into the WT PDXK 3′UTR to abolish the predicted pairing sit of miR-339. The verification of PDXK 3′UTR mutant vector and mutant sequence map are provided in Supplementary Figures 3, 4. Generally, luciferase activity was notably downregulated in the PDXK-WT + miR-339 group in comparison to that in the PDXK-WT + NC group. Additionally, these PDXK mutants have revealed that the luciferase activity was highly reduced in PDXK 3′UTR WT group and slightly restored in the Mut2, Mut3, and Mut4 groups (^∗^*P* < 0.05) (Figures 4C-d,e,f).

### MiR-339 Inhibition Could Promote the Neurite Growth and Decrease Neuronal Apoptosis Associated With PDXK Upregulating

**FIGURE 5:**
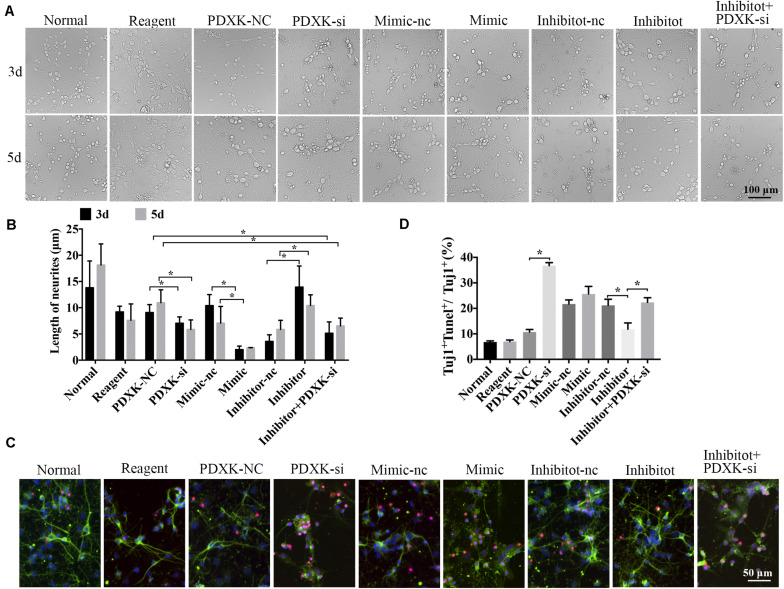
The role of miR-339 on neurite growth. **(A)** The cell physiological status under different situation in normal, reagent, NC, mimic-NC, inhibitor-NC, mimic, inhibitor, PDXK-si, and inhibitor + PDXK-si groups on 3 and 5 days after transfection. Scale bar = 100 μm. **(B)** Quantitative results of the length of neurites in normal, reagent, NC, mimic-NC, inhibitor-NC, mimic, inhibitor, PDXK-si, inhibitor + PDXK-si groups at 3 and 5 days post-transfection. **(C)** Evaluation of apoptotic cortical neurons by Tuj1 immunofluorescent staining and TUNEL staining, presented by Tuj1 + Tunel + /Tuj1 + (%) in normal, reagent, NC, mimic-NC, inhibitor-NC, mimic, inhibitor, PDXK-si, inhibitor + PDXK-si groups. DAPI counterstaining (blue) shows nuclei of intact cells, green fluorescence represented Tuj1 +, and red fluorescence represented apoptosis. Scale bar = 50 μm. **(D)** Quantitative analysis for neuronal apoptosis, presented by Tuj1 + Tunel + /Tuj1 + (%) in normal, reagent, NC, mimic-NC, inhibitor-NC, mimic, inhibitor, and PDXK-si, inhibitor + PDXK-si groups. Data were exhibited as mean ± SD; *P < 0.05. PDXK, pyridoxal kinase; NC, negative control; PDXK-si, PDXK interference; TUNEL, terminal deoxyribonucleotidyl transferase-mediated dUTP-digoxigenin nick-end labeling; DAPI, 4,6-diamidino-2-phenylindole.

Morphological changes of neurons were observed through a Leica AF6000 cell station (CM8600, Leica Microsystems, Buffalo Grove, IL, United States) at 3 and 5 days post-transfection under bright-field microscopy (Figure 5A). Quantitative analysis has shown that the length of neurites was significantly raised in the miR-339 inhibitor group in comparison to that in the inhibitor-nc group at 3 and 5 days after transfection, while the neuron growth was partially weakened by PDXK-si (^∗^*P* < 0.05) (Figure 5B). Reversely, in comparison to the mimic-nc group, the length of neurites were clearly decreased in the miR-339 mimic group (^∗^*P* < 0.05) (Figure 5B). In addition, these parameters were greatly decreased in PDXK-si group and PDXK-si + inhibitor group in comparison to those in the NC group (^∗^*P* < 0.05) (Figure 5B). To detect the role of miR-339/PDXK-si transfection on neuronal apoptosis, apoptotic rate of neurons was evaluated using TUNEL staining. Neurons with PDXK-si transfection have exhibited higher number of apoptotic neurons than the NC group (^∗^*P* < 0.05) (Figures 5C,D), while the apoptotic neurons in the miR-339 inhibitor group were obviously decreased compared with NC group (^∗^*P* < 0.05) (Figures 5C,D).

### MiR-339 Knockout Promotes the Neurite Growth and Functional Recovery, as Well as Decreased Neuronal Apoptosis via Upregulating the Expression Level of PDXK

**FIGURE 6:**
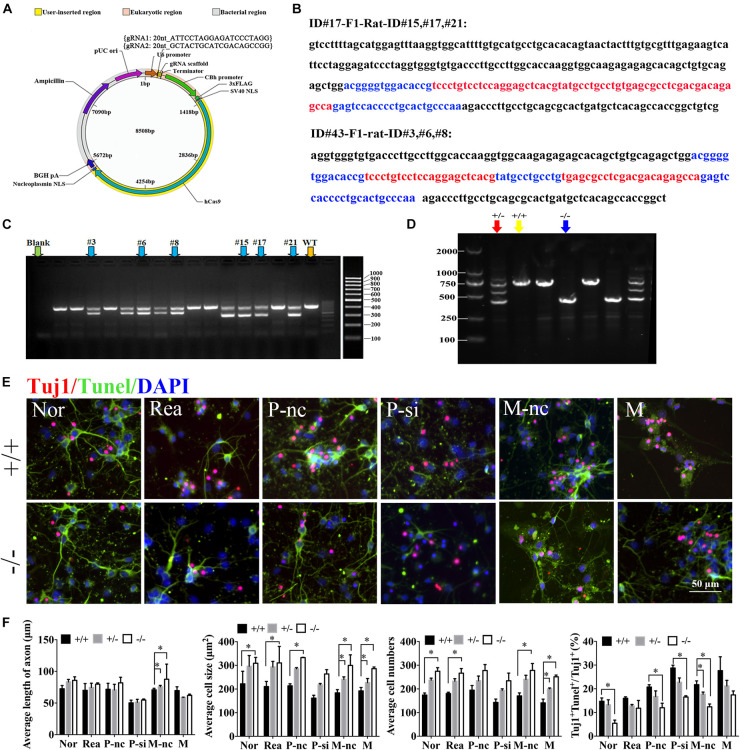
The role of miR-339 and its regulatory relationship with PDXK on neurite growth at miR-339 knockout neurons. **(A)** The construction of vector. **(B)** Sequencing results: the deleted sequence is black, and the exon sequence is highlighted in blue and red. **(C)** F1 founder PCR screening: the molecular weight of the wild-type rats is 729 bp; the missing molecular weight of #15, #17, and #21 is 300 bp; the missing molecular weight of #3, #6, and #8 is 202 bp. **(D)** Electrophoretic band chart for genotype detection. Red green arrow refers to heterozygote rats, yellow arrow refers to wild-type rats, and blue arrow refers to knockout rats. The markers exhibit 100, 250, 500, 750, 1,000, and 2,000 bp, respectively. ± represents heterozygote rats, +/+ represents wild-type rats, and −/− represents the knockout rats. **(E)** Photomicrographs of neurons detected by Tuj1 staining in Nor, Rea, P-nc, P-si, M-nc and M groups of −/− and +/+ neurons. DAPI counterstaining (blue) demonstrated nuclei of intact cells, and red fluorescence represented Tuj1 positive staining. Apoptotic neurons were determined by Tuj1 and TUNEL staining, presented by Tuj1 + Tunel +/Tuj1 + (%) in Nor, Rea, P-nc, P-si, M-nc and M groups of −/− and +/+ neurons. DAPI counterstaining (blue) demonstrated nuclei of intact cells, red fluorescence represented Tuj1, and red fluorescence represented apoptosis. Scale bar = 50 μm. **(F)** The length of axon, cell size, cell numbers and apoptosis rate in Nor, Rea, P-nc, P-si, M-nc and M groups of −/−, +/− and +/+ neurons. DAPI counterstaining (blue) demonstrated nuclei of intact cells, red fluorescence represented Tuj1, and red fluorescence represented apoptosis. Scale bar = 50 μm. Data were exhibited as mean ± SD. *p < 0.05 vs. +/+, ^#^*p* < 0.05 vs. P-nc. Nor, normal; Rea, reagent; P-nc, PDXK-nc; P-si, PDXK-si; M-nc, miR-339-mimic-nc; M, miR-339-mimic; WT, wild type; NC, negative control. −/− represents miR-339 knockout homozygote. +/+ represents wild type. ± represents heterozygote. TUNEL, terminal deoxyribonucleotidyl transferase-mediated dUTP-digoxigenin nick-end labeling; DAPI, 4,6-diamidino-2-phenylindole.

To further confirm the role of miR-339 and its regulatory relationship with PDXK in function, miR-339 knockout rats were constructed via CRISPR-caspase9 technology. The sgRNA vector was successfully constructed by sequencing to construct miR-339 knockout rats (Figure 6A). Next, the rats target amplification product of gene modification in F1 generation under F0 generation was performed. The genetic modification of CRISPR/Cas9 on miR-339 was determined by TA cloning and sequencing. The sequencing results showed the deleted sequence in black, and the exon sequence is highlighted in blue and red (Figure 6B). For F0 founder PCR screening, the molecular weight of the wild-type rats is 729 bp, the missing molecular weight of #15, #17, and #21 is 300 bp, and the missing molecular weight of #3, #6, and #8 is 202 bp (Figure 6C). After administrating the microinjection to F0, we began to reproduce to acquire F1. To verify the genetic modification of miR-339, PCR and gel electrophoresis were conducted to determine heterozygote (HE), WT, and homozygote (KO) off-springs. After detection, the animals were used for the later experiment (Figure 6D). The neurons from miR-339 WT (+ / +) or homozygous knockout rats (−/−) were identified by Tuj1 staining. Then, the growth situation of cortical neurons and cell apoptosis were studied under the fluorescent microscope (Figures 6E,F). Quantitative analysis showed that the cell size and cell numbers from −/− neurons were obviously increased in normal (Nor), regent (Rea), mimic-nc (M-nc), and mimic (M) groups when compared with those in + / + neurons (^∗^*P* < 0.05) (Figure 6G). In addition, the length of axon from −/− and heterozygote (±) neurons were significantly increased in the M-nc group in comparison to + / + (^∗^*P* < 0.05) (Figure 6G). The cell size of −/− was also distinctly increased in P-nc when compared with + / + (^∗^*P* < 0.05) (Figure 6G). Furthermore, the apoptosis rate from + / + neurons in Nor, P-nc, P-si, and M-nc groups was higher than those from −/− neurons (^∗^*P* < 0.05) (Figure 6G); however, the effect of decreasing apoptosis rate was partly reversed by PDXK-si when compared with PDXK-nc group (^#^*P* < 0.05 vs. P-nc) (Figure 6G).

Moreover, the BBB score of miR-339 knockout rats was significantly increased at 14 and 28 days when compared with control group (^∗^*P* < 0.05; ^∗∗^*P* < 0.01) (Supplementary Figure 5A). In addition, through GeneMANIA^7^, there is a coexpression network between PDXK and GAP43. As GAP43 has been designated as a last downstream molecule for axonal regeneration, the link between PDXK and GAP43 indicates that PDXK may function with GAP43 for axonal regeneration, and PDXK improving the neurological function may cooperate with GAP43 (Supplementary Figure 5B).

## Discussion

In this study, we demonstrated that PDXK is expressed in the remote motor cortex of the spinal cord in rats with SCT. PDXK overexpression and interference *in vitro* influence neuronal survival and neuronal growth and improve or inhibit motor function in rats with SCT, respectively. Moreover, miR-124 or miR-339 was found to regulate 3′UTR of PDXK mRNA, whereas by luciferase and the mutant detection test, we found that miR-339 but not miR-124 completely match with the position of 3′UTR in PDXK to inhibit the translation of PDXK. Neuronal phenotypes from the miR-339 knockout rat reported the vital function for miR-339 in the cell experiment, indicating that miR-339 is crucial to PDXK/GAP43 in functional remodeling in SCT rats.

First, we established a SCT model, and a behavior test was used to test the neurological deteriorates induced by SCT (Cen et al., 2013; M’dahoma et al., 2014; Li et al., 2015), as it is well known that the motor center is located in the cerebral cortex. The spinal cord has a low-level body movement center that is regulated by the high-level motor center of the cerebral cortex. The anterior horn of the spinal cord is responsible for motor function, and its damage can lead to motor dysfunction. The BBB score, which is a valid and predictive measure, was used to test motor function after SCT, which can also indicate motor recovery (Rossignol et al., 1999; He et al., 2016). This study also focused on motor function recovery after SCI, and therefore, cortical neurons were selected as *in vitro* experimental subjects because motor neurons originate in the cerebral cortex (Wang et al., 2016).

By thoroughly investigating the molecules involved in functional remodeling of the spinal cord in remote cortexes, we discovered that PDXK is substantially upregulated in the motor cortex after SCT. It has been reported that the function of PDXK is prominently enhanced in the whole brain tissue of humans, embryos, and fetuses (Bukin Iu and Ivanova, 1976; Shin et al., 2004), as well as in the whole brain of in epilepsy-prone and epilepsy-resistant rats (Ebadi et al., 1985). Additionally, PDXK is expressed in the porcine brain cortex (Gao et al., 1998). PDXK is also expressed in the cytoplasm of neuronal and neuroglial cells in the cerebral cortex, hippocampus, nuclei, and cerebellar cortex in rabbits (Ohkawa et al., 1994). These indicate that PDXK plays a vital role in the nervous system. However, the correlation of PDXK and SCT is unclear. Thus, our study provided a novel sight for understanding the mechanism of PDXK in SCT.

In addition, we have determined the function of PDXK *in vitro* and *in vivo* by overexpressing and inhibiting PDXK. All results showed that PDXK overexpression in the cortex promotes motor function recovery and neuron survival and decreases cell apoptosis. A previous study demonstrated that vitamin B6 reduces the risk of cancer by restricting genome rearrangements, which was correlated with the stimulation of the DNA damage. Thus, PDXK increases genome stability by enhancing the metabolism of dietary vitamin B6 into its biologically active derivative, pyridoxal-5′-phosphate (Kanellis et al., 2007). In addition, PDXK participates in the conversion of pyridoxine and other vitamin B6 precursors into PLP. Therefore, it was shown to be a prognostic factor in patients with lung carcinoma (Galluzzi et al., 2013). Moreover, upregulating the expression of PDXK had an apparently positive impact on the overall survival of non-small cell lung cancer patients (Galluzzi et al., 2012; Aranda et al., 2014). In addition, it was indicated that human PDXK could be suppressed by drugs, such as theophylline and progabide, which are associated with several adverse events such as vitamin B6 deficiency and central nervous system-related abnormalities (Laine-Cessac et al., 1997). Undoubtedly, PDXK is connected with functions of the nervous system, as several neurotransmitters like dopamine, norepinephrine, serotonin, and γ-aminobutyric acid are synthesized by pyridoxal 5′-phosphate-dependent enzymes (Dakshinamurti et al., 1990). Disorders of metabolism of PDXK activity can lead to disorders of amino acid metabolism, which may be the pathogenesis of secondary and other nervous system damage. Until now, the function of PDXK in the cortex of rats after SCT was not investigated. It is therefore the first time that the role of PDXK in the remote control of cerebral cortex in SCT rats has been demonstrated.

Moreover, the present study demonstrates that miR-339 is a complete match with the position of 3′UTR in PDXK and regulates PDXK expression. We have also found that neurons from miR-339 knockout rats exhibit apparent neuronal outgrowth and decreased cell apoptosis, while these effects were partly reversed through PDXK interference, indicating that there is a functional regulatory relationship between miR-339 and PDXK. A previous study reported that miR-339 (-5p) directly targeted the 3′UTR of murine double minute 2 (MDM2) mRNA to reduce MDM2 expression and promote p53 function (Jansson et al., 2015). In addition, miR-339 decreases the mu-opioid receptor (MOR) by modulating its 3′UTR in response to opioid treatment (Wu et al., 2013). Moreover, miR-339 regulates the metastasis, growth, and colony formation of colorectal cancer cells by targeting 3′UTR of phosphatases of regenerating liver-1 mRNA (Zhou et al., 2013). Comparatively, our study is the first to prove the role that is played by miR-339 in negatively regulating PDXK expression in the cortex of SCT rats.

In this study, we also noticed that PDXK is associated with GAP43 from GeneMANIA bioinformatics prediction, which may underlie the mechanism for nerve regeneration and functional improvement in the cortex of SCT rats. GAP43 is an axonal membrane protein confirmed molecule that is an important part of the regulation mechanism of cortical axon pathfinding (Denny, 2006; Shen and Meiri, 2013). In particular, it is involved in extracellular growth and synapse formation and promotes nerve regeneration, thereby mediating neuroprotective effects. The high expression of GAP43 gene is considered to be a typical feature of neural regeneration and is closely related to spinal cord regeneration (Bekar et al., 2014; Liu et al., 2014). This study outlines the mechanism behind functional improvement in the remote cortex of the spinal cord with SCT, which can be explained by miR-339 targeting PDXK associated with the GAP43 to promote nerve regeneration and functional improvement in SCT rats. These crucial findings provide a novel idea for understanding the molecular events in the remote cortex after spinal cord injury.

**FIGURE 7:**
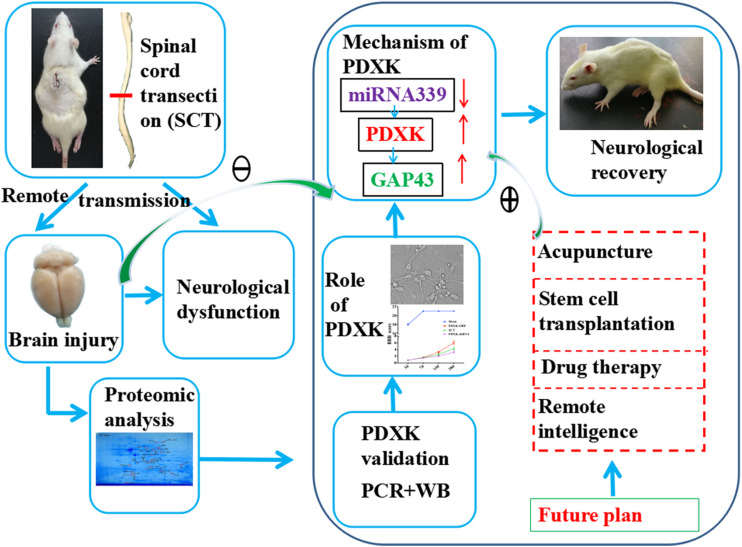
The underlying mechanism that how PDXK promoted neurological recovery of SCT rats. First, the SCT model was successfully established, and the brain injury and neurological dysfunction were observed. By 2D electrophoresis and MALDI-TOF-MS, we found that PDXK was downregulated at 3 days and upregulated at 14 and 28 days after SCT. Then, it was confirmed that PDXK was expressed in cortical neurons via double immunofluorescent labeling, which indicated that PDXK may exert a crucial role in functional recovery in SCT rats. Next, it was confirmed that the BBB score and the neurons were all significantly increased under the treatment of PDXK-ORF. Afterward, the prediction of “miRwalk,” “miRDB,” “miRanda,” and “TargetScan” revealed that PDXK was a target gene of miR-339; meanwhile, it was pointed that PDXK was specifically regulated by miR-339. Through inhibiting the expression of miR-339, it was affirmed that the cell size, the number of neurons, and the axon length were all increased. Finally, the GAP43 was screened out as the mechanism pathway by gene MANIA. For the future plan, the treatment of acupuncture, stem cell transplantation, drug therapy, and remote intelligence may perform to downregulate the expression of miR-339 and upregulate the expression of PDXK and GAP43 to promote neurological recovery. SCT, spinal cord transection; GAP43, growth associated protein-43; BBB, Basso, Beattie, and Bresnehan.

In conclusion, we have identified that PDXK is important in the motor cortex of SCT rats, in which, PDXK overexpression improves significantly motor function, increases neuronal activity, and reduces neuronal apoptosis. Moreover, miR-339 can target PDXK, which influences functional remodeling in the remote cortex of SCT rats. These crucial findings may contribute to the understanding of miR-339 regulating PDXK/GAP43 signal in the remote cortex after SCT and give an indication about how they can be applied in future clinic trials (Figure 7).

## Data Availability Statement

The mass spectrometry proteomics data have been deposited to the ProteomeXchange via the PRIDE database. Data are available via ProteomeXchange with identifier PXD017900.

## Ethics Statement

The animal study was reviewed and approved by the Institutional Animal Care and Use Committee of Kunming University (kmmu2019005).

## Author Contributions

L-LXi, XB, T-HW, Y-CW, VB, and X-FZ participated in the experimental conception and design. L-LXu, Y-XQ, Y-CW, YJ, ZM, and JL performed the experiments. L-LXu, Y-XQ, XB, JL, and MA-H performed the data collection and analysis. L-LXu, R-LD, MA-H, and Q-XX wrote the initial manuscript. L-LXi and T-HW revised the article. All authors contributed to draft and read the article as well as approved the final version.

## Conflict of Interest

The authors declare that the research was conducted in the absence of any commercial or financial relationships that could be construed as a potential conflict of interest.

## Footnotes

1
www.cyagen.com

2
http://www.targetscan.org/vert_72/docs/help.html

3
http://www.mirdb.org

4
www.microrna.org

5
http://mirwalk.umm.uni-heidelberg.de

6
http://bioinfogp.cnb.csic.es/tools/venny/index.html

7
http://genemania.org/
