# Corrigendum: MicroRNA339 Targeting PDXK Improves Motor Dysfunction and Promotes Neurite Growth in the Remote Cortex Subjected to Spinal Cord Transection

**DOI:** 10.3389/fcell.2022.877291

**Published:** 2022-04-13

**Authors:** Liu-Lin Xiong, Yan-Xia Qin, Qiu-Xia Xiao, Yuan Jin, Mohammed Al-Hawwas, Zheng Ma, You-Cui Wang, Visar Belegu, Xin-Fu Zhou, Lu-Lu Xue, Ruo-Lan Du, Jia Liu, Xue Bai, Ting-Hua Wang

**Affiliations:** ^1^ Institute of Neurobiological Disease, Department of Anesthesiology, Translational Neuroscience Center, West China Hospital, Sichuan University, Chengdu, China; ^2^ Animal Zoology Department, Institute of Neuroscience, Kunming Medical University, Kunming, China; ^3^ National Traditional Chinese Medicine Clinical Research Base and Western Medicine Translational Medicine Research Center, Department of Cardiac and Cerebral Diseases, Department of Anesthesiology, Affiliated Traditional Chinese Medicine Hospital, Southwest Medical University, Luzhou, China; ^4^ School of Pharmacy and Medical Sciences, Sansom Institute, Division of Health Sciences, University of South Australia, Adelaide, SA, Australia; ^5^ Department of Histology and Neurobiology, College of Preclinic and Forensic Medicine, Sichuan University, Chengdu, China; ^6^ International Center for Spinal Cord Injury, Kennedy Krieger Institute, Baltimore, MD, United States; ^7^ Department of Neurology and Pathology, Johns Hopkins University School of Medicine, Baltimore, MD, United States

**Keywords:** microRNA339, motor cortex plasticity, PDXK, RNA interference, spinal cord injury

In the original article, there was a mistake in Figure 6A as published. In the original Figure 6A, “there was only sequence of one gRNA, but the sequences of two gRNAs should be provided in the vector map for knocking out microRNA-339, thus we have updated the sequences of two gRNAs in the corrected Figure 6A.” The corrected Figure 6A appears below.

**FIGURE 6:**
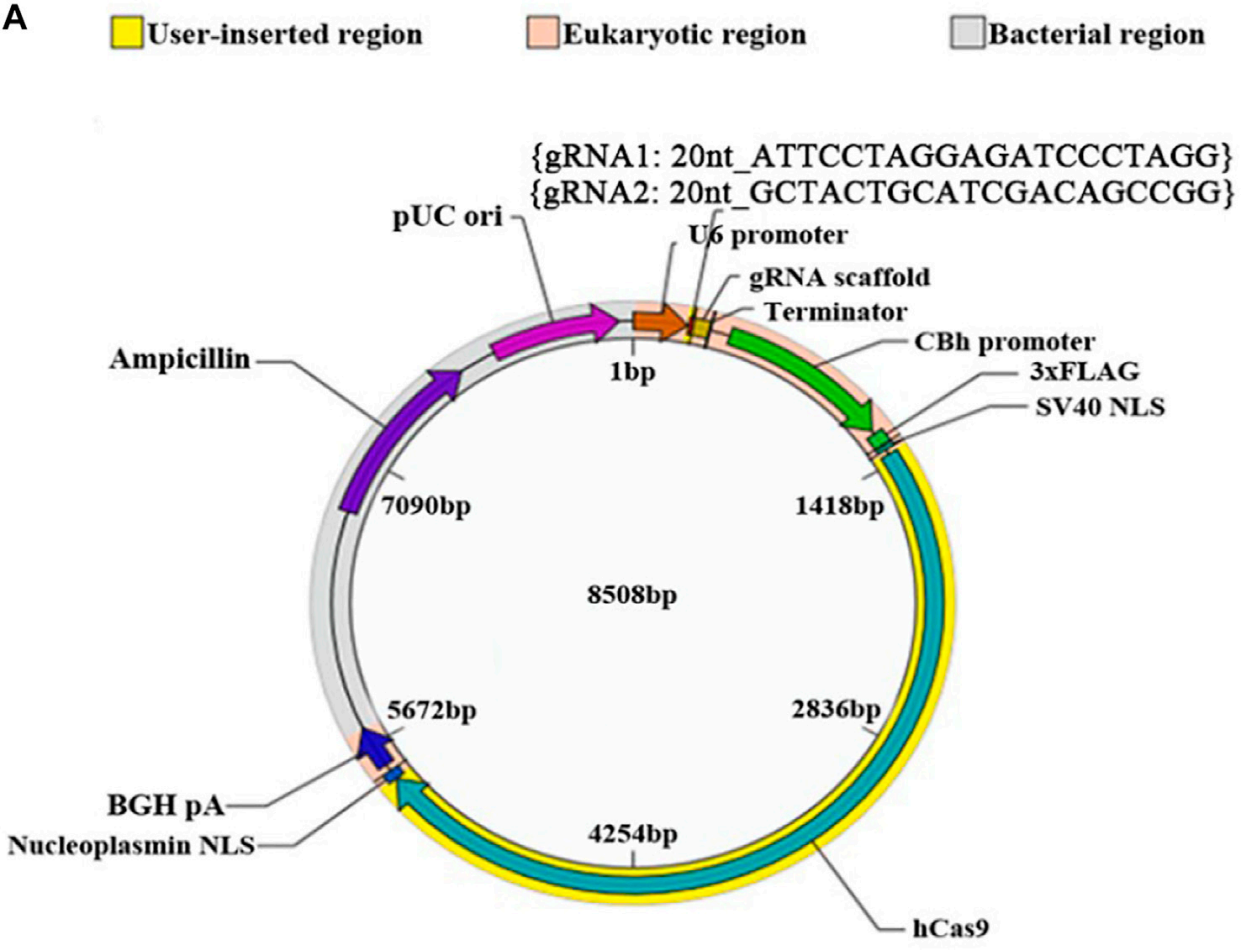
The role of miR-339 and its regulatory relationship with PDXK on neurite growth at miR-339 knockout neurons. **(A)** The construction of vector. **(B)** Sequencing results: the deleted sequence is black, and the exon sequence is highlighted in blue and red. **(C)** F1 founder PCR screening: the molecular weight of the wild-type rats is 729 bp; the missing molecular weight of #15, #17, and #21 is 300 bp; the missing molecular weight of #3, #6, and #8 is 202 bp. **(D)** Electrophoretic band chart for genotype detection. Red green arrow refers to heterozygote rats, yellow arrow refers to wild-type rats, and blue arrow refers to knockout rats. The markers exhibit 100, 250, 500, 750, 1,000, and 2,000 bp, respectively. ± represents heterozygote rats, +/+ represents wild-type rats, and −/− represents the knockout rats. **(E)** Photomicrographs of neurons detected by Tuj1 staining in Nor, Rea, P-nc, P-si, M-nc and M groups of −/− and +/+ neurons. DAPI counterstaining (blue) demonstrated nuclei of intact cells, and red fluorescence represented Tuj1 positive staining. Apoptotic neurons were determined by Tuj1 and TUNEL staining, presented by Tuj1 + Tunel +/Tuj1 + (%) in Nor, Rea, P-nc, P-si, M-nc and M groups of −/− and +/+ neurons. DAPI counterstaining (blue) demonstrated nuclei of intact cells, red fluorescence represented Tuj1, and red fluorescence represented apoptosis. Scale bar = 50 μm. **(F)** The length of axon, cell size, cell numbers and apoptosis rate in Nor, Rea, P-nc, P-si, M-nc and M groups of −/−, +/− and +/+ neurons. DAPI counterstaining (blue) demonstrated nuclei of intact cells, red fluorescence represented Tuj1, and red fluorescence represented apoptosis. Scale bar = 50 μm. Data were exhibited as mean ± SD. *p < 0.05 vs. +/+, ^#^ *p* < 0.05 vs. P-nc. Nor, normal; Rea, reagent; P-nc, PDXK-nc; P-si, PDXK-si; M-nc, miR-339-mimic-nc; M, miR-339-mimic; WT, wild type; NC, negative control. −/− represents miR-339 knockout homozygote. +/+ represents wild type. ± represents heterozygote. TUNEL, terminal deoxyribonucleotidyl transferase-mediated dUTP-digoxigenin nick-end labeling; DAPI, 4,6-diamidino-2-phenylindole.

The authors apologize for this error and state that this does not change the scientific conclusions of the article in any way. The original article has been updated.

## Supplementary Material

Supplementary File 1 The sequence of gRNA1 in the vector map for knocking out microRNA-339.

Supplementary File 2 The sequence of gRNA2 in the vector map for knocking out microRNA-339.

