# Supplementary material for: MicroRNA339 Targeting PDXK Improves Motor Dysfunction and Promotes Neurite Growth in the Remote Cortex Subjected to Spinal Cord Transection
Source: Front Cell Dev Biol. 2020 Jul 21;8:577. doi: 10.3389/fcell.2020.00577 (PMC7386314; doi:10.3389/fcell.2020.00577)
Supplement: Supplementary file 1 [file Data_Sheet_1.pdf]

Supplementary Material

1 Supplementary Tables

Supplementary Table 1. Preparation of animal model and sample used.

Groups	Treatment	Proteomics	WB/PCR
Sham	Sham operation	5	6/6
3dpo	SCT	5	6/0
14dpo	SCT	5	6/0
28dpo	SCT	5	6/6

SCT: Spinal cord transection; dpo: day post-operation; IF: Immunofluorescent staining; WB: Western Blot.

Supplementary Table 2. Animal grouping and number in each test.

Groups	Treatment	BBB score	IF/WB
Sham	Sham operation	9	3/6
Negative-control	SCT+Lv-NC vector	9	3/6
PDXK ORF	SCT+ EX-Rn10146-Lv201	9	3/6

PDXK shRNA	SCT+ RSH051349-HIVmU6	9	3/6
miR-339 KO	miR-339 knock out	9	0/0

BBB: Basso, Beattie, and Bresnehan; NC: Negative-control; SCT: Spinal cord transection; PDXK: Pyridoxal kinase.

Supplementary Table 3. The details about the PDXK siRNA are showed as follows.

Name	Catalog No	Sense strand	Antisense strand
Si-r-PDXK_001	siG09102883915	5´GCAUGGUGGUGGAUAUCGU dTdT 3´	3´dTdT CGUACCACCACCUAUAGCA 5´
Si-r-PDXK_002	siG09102883902	5´ GCAAACAAUGUCAACAAGU dTdT3´	3´dTdT CGUUUGUUACAGUUGUUCA 5´
Si-r-PDXK_003	siG09102883848	5´AGAACUCUCGACUCGUGUA dTdT 3´	3´dTdT UCUUGAGAGCUGAGCACAU 5´

PDXK: Pyridoxal kinas; No: Number; siRNA: small interference.

Supplementary Table 4. Primers used in this experiment

Gene	Primers
PDXK	PDXK-3UTR-F:5'<br>CCGCTCGAGTGCCTTAGAGCCATGACTGAAAC 3'<br>PDXK-3UTR-R:5' GAATGCGGCCGCCTGGTGGATACTGAAACTG<br>3'
PDXK-Mut-2	m-PDXK-mut-F610: 5'<br>GTTGGACACAGACTCAAGGAGAGACAGAGGT 3'<br>m-PDXK-mut-R632: 5'

	TTAAAAGGTGAGTCTGACAGTGACCGCAT 3'
PDXK-Mut-3	m-PDXK-mut-F1026:5' TTGGACACAGACTCAAGGAGAGACAGAGGT 3'<br>m-PDXK-mut-R1047:5' TCTCTCCTTGAGTCTGTGTCCAACCCCCTGTG 3'
PDXK-Mut-4	m-PDXK-mut-F610: 5' GTTGGACACAGACTCAAGGAGAGACAGAGGT 3'<br>m-PDXK-mut-R1047:5' TCTCTCCTTGAGTCTGTGTCCAACCCCCTGTG 3'

PDXK: Pyridoxal kinas.

Supplementary Table 5. Differential proteins identified by spectrum analysis.

Spots	Protein Name	Accession GI	PI/MW (Observed)	PI/MW (Calculated)	Score	Sequence Coverage
1	Carbonic anhydrase 1	62643144	6.86/28282	6.05/26860	374	69%
3	Protein carboxyl methyl transferase	603467	7.14/24610	6.14/22120	167	46%
4	Rieske Fe-S protein percursor	206681	8.90/27671	6.05/22270	432	31%

5	NADH dehydrogenase	27661165	5.87/23955	5.39/17680	177	53%
6	Glutamine synthetase	121376	6.64/42240	6.35/39640	320	30%
7	Endoplasmic retuclum protein 29	16758848	6.23/28557	5.68/15010	254	38%
9	Peroxiredoxin 2	8394432	5.34/21770	5.39/14710	109	31%
10	α-GDP dissociation inhibitor	31982030	5.12/23393	5.38/21820	237	50%
11	Superoxide dismutase 2	8394331	8.96/24659	7.36/28270	377	49%
17	α-synuclein	9507125	5.74/14506	5.17/39000	363	45%
18	LIM and SH3 protein 1	14249130	8.96/24659	7.36/28270	237	50%
19	Glutamate dehydrogenase 1	6980956	8.05/61377	8.95/58120	182	21%
20	Glutamine synthetase	228136	6.38/40411	5.86/51450	301	24%
21	Dihydropyrimidinase-2	40254595	5.95/62239	5.72/66120	326	25%

22	Pridoxa kinase	13929082	6.32/34886	5.69/39300	258	31%
23	Aldose reductase 1	6978491	6.26/35774	5.81/40930	258	27%
24	Annexin Ⅴ	1421099	4.97/35401	5.27/35160	394	40%
25	Pyruvate kinase	16757994	6.63/57781	5.93/65520	292	26%
28	4-aminobutyrate aminotransferase	122065191	8.15/56419	6.11/55600	200	20%
29	Aconitase 2	40538860	7.87/85380	7.16/75450	290	18%
30	3-oxoacid coa transferase 1	109466092	8.70/56168	7.07/61520	349	29%
31	Transferrin	61556986	7.14/76346	6.99/75150	60	12%
32	Purine-nucleoside phosphorylase	34869683	6.46/32281	5.81/30860	283	44%
33	Synapsin Ⅰ 1	33357051	6.22/35014	5.91/61380	62	7%
34	Malate dehydrogenase 1	15100179	6.16/36460	5.62/41080	164	12%

35	Actin binding protein 1A	18426834	6.05/51033	5.75/63600	49	8%
36	β-Guanine nucleotide-binding protein	13937391	5.60/37307	5.50/39160	87	12%
37	Aconitase 2	40538860	7.87/85380	7.16/75460	245	16%

6 and 20 are the same protein, 29 and 37 are the same protein that was identified in different gel which indicate that tandem mass spectrometry identification technology is reliable in this experiment.

Supplemental mass spectrum data 1

PMF

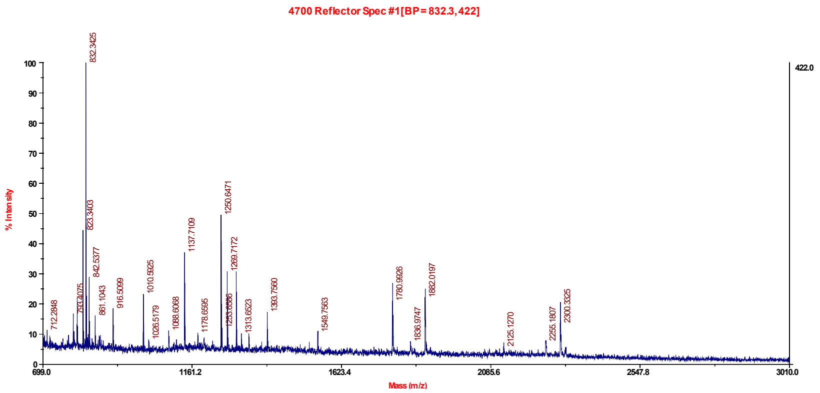

MSMS

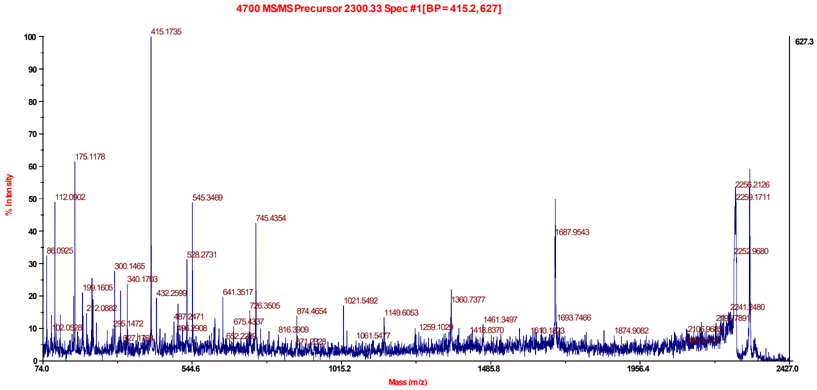
4700 MS/MS Precursor 2300.33 Spec #1[BP = 415.2, 627]
415.1735
175.1178
112.0902
545.3469
745.4354
1687.9543
2256.2126
2259.1711
2252.9680
86.0925
528.2731
300.1465
340.1703
199.1605
432.2599
641.3517
1360.7377
1021.5492
726.3505
874.4654
1149.6053
1693.7466
1874.9082
% Intensity
Mass (m/z)
627.3

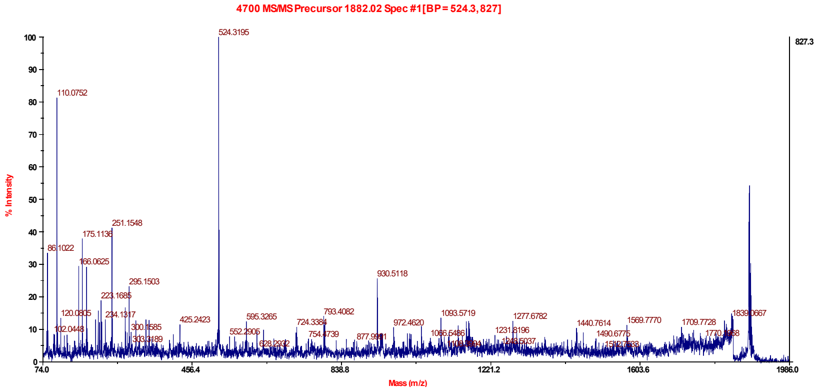
4700 MS/MS Precursor 1882.02 Spec #1[BP = 524.3, 827]
524.3195
110.0752
251.1548
175.1136
86.1022
166.0625
930.5118
295.1503
223.1685
120.0805
234.1317
425.2423
595.3265
793.4082
724.3384
1093.5719
1277.6782
1440.7614
1569.7770
1709.7728
972.4620
% Intensity
Mass (m/z)
827.3

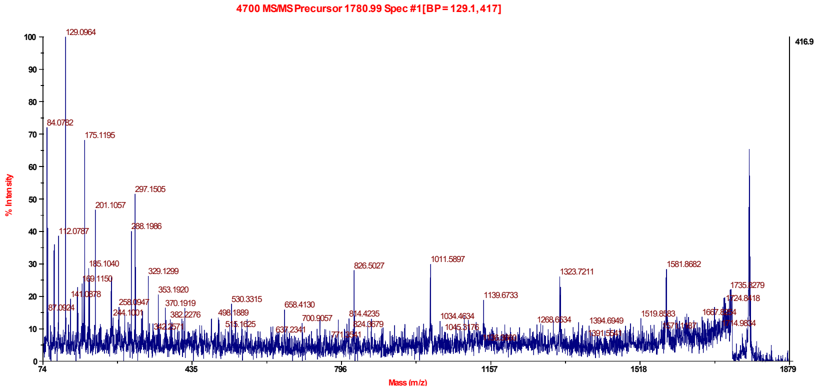
4700 MS/MS Precursor 1780.99 Spec #1[BP = 129.1, 417]
129.0964
84.0782
175.1195
297.1505
201.1057
288.1986
112.0787
185.1040
329.1299
826.5027
1011.5897
1323.7211
1581.8682
353.1920
370.1919
530.3315
658.4130
1139.6733
1034.4634
1394.6949
1519.8583
% Intensity
Mass (m/z)
416.9

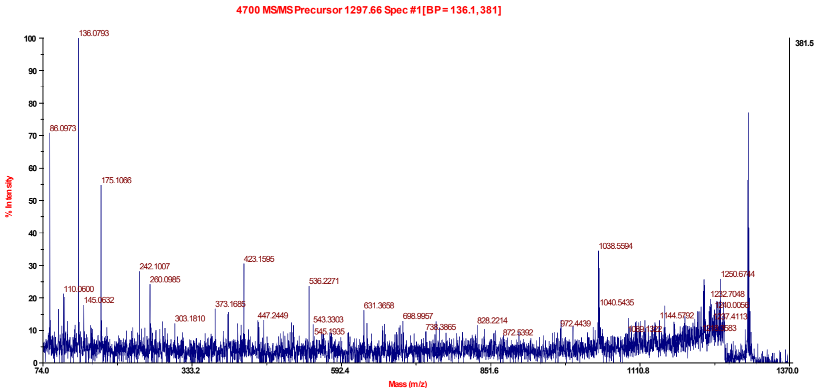

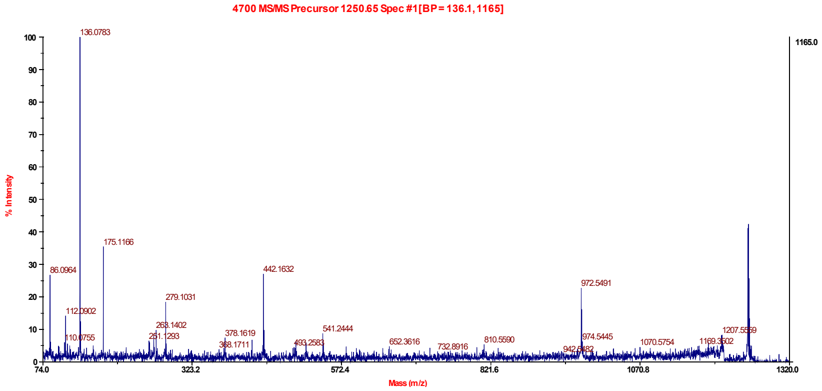

Mass Spectrometry information of PDXK (NO.22 spot)

Table S6 list the potential protein that identified by Mass Spectrometry on 4700 Proteomics Analyzer. Pyridoxal kinase (PDXK), which the Probability Based Mowse Score was 258 (greater than 56 means significant), was accepted; Table S7 shows 12 peptides of PDXK were identified by Mass Spectrometry. The peptide mass fingerprinting (PMF) is as follows. Matched and no matched peaks are also listed in Table S6. Tandem mass spectrometry (MS/MS) confirmed the “2300.33” peptide (140-160), “1882.02” peptide (162-177), “1780.99” peptide (276-292), “1297.66” peptide (195-206) and 1250.65 peptide (77-86); and the ions score is 56, 40, 28, 23, 29, respectively. For each PMF and MS/MS picture, we could use mouse to drag the pictures to their clearly size.

Supplementary Table 6. List of potential protein (No.1 was accepted).

NO	Accession	Mass	Score	Description

1	gi|13929082	34886	258	pyridoxal (pyridoxine, vitamin B6) kinase
2	gi|62665802	34890	255	PREDICTED: similar to pyridoxal (pyridoxine, vitamin B6) kinase
3	gi|9506507	53811	38	coronin, actin-binding protein, 1B
4	gi|28381370	11564	37	Protein KIAA1688 homolog (Preoptic regulatory factor 2) (PORF-2)
5	gi|539953	21207	32	enhancer factor I chain A-D
6	gi|559637	4391	30	nuclear orphan receptor HZF-1=glucocorticoid/mineralocorticoid receptor homolog
7	gi|11321107	5912	28	p53 tumor suppressor
8	gi|66730402	10119	28	p300/CBP-associated factor
9	gi|8307696	20817	27	alpha-2u globulin
10	gi|57528321	62681	27	RIO kinase 2

Supplementary Table 7. The details about the PDXK are showed as follows.

**Observed**	**Mr(expt)**	**Mr(calc)**	**Delta**	**Start - End**	**Miss**	**Ions**	**Peptide(**Matched peptides shown in Bold Red**)**	[illegible]
1137.71	1136.70	1136.67	0.04	7-16	0	---	R.VLSIQSHVVR.G	Y
1250.65	1249.64	1249.60	0.04	77-86	0	29	K.YDYVLTGYTR.D	

1250.65	1249.64	1249.60	0.04	77-86	0	---	K.YDYVLTGYTR.D	Y
1269.72	1268.71	1268.67	0.04	259-269	0	---	K.TVSAMQHVLQR.T	YES
1297.66	1296.66	1296.61	0.04	195-206	0	23	K.GSDYLMALGSQR.M	
1297.66	1296.66	1296.61	0.04	195-206	0	---	K.GSDYLMALGSQR.M	Y
1780.99	1779.99	1779.91	0.07	276-292	0	28	K.AEAGEGQKPSPAQLELR.M	
1780.99	1779.99	1779.91	0.07	276- 292	0	---	K.AEAGEGQKPSPAQLELR.M	Y
1882.02	1881.01	1880.92	0.09	162-177	0	40	K.IHSQEEAFAVMDVLHR.M	
1882.02	1881.01	1880.92	0.09	162-177	0	---	K.IHSQEEAFAVMDVLHR.M	Y
2300.33	2299.33	2299.19	0.14	140-160	0	56	K.VVPMADIITPNQFEAELLSGR	
2300.33	2299.33	2299.19	0.14	140-160	0	---	K.VVPMADIITPNQFEAELLSGR.K	YI

PDXK: Pyridoxal kinas;

No match to: 793.41, 804.31, 805.54, 823.34, 832.34, 861.10, 916.51, 1010.59, 1393.75, 1549.75

2 Supplementary Figures

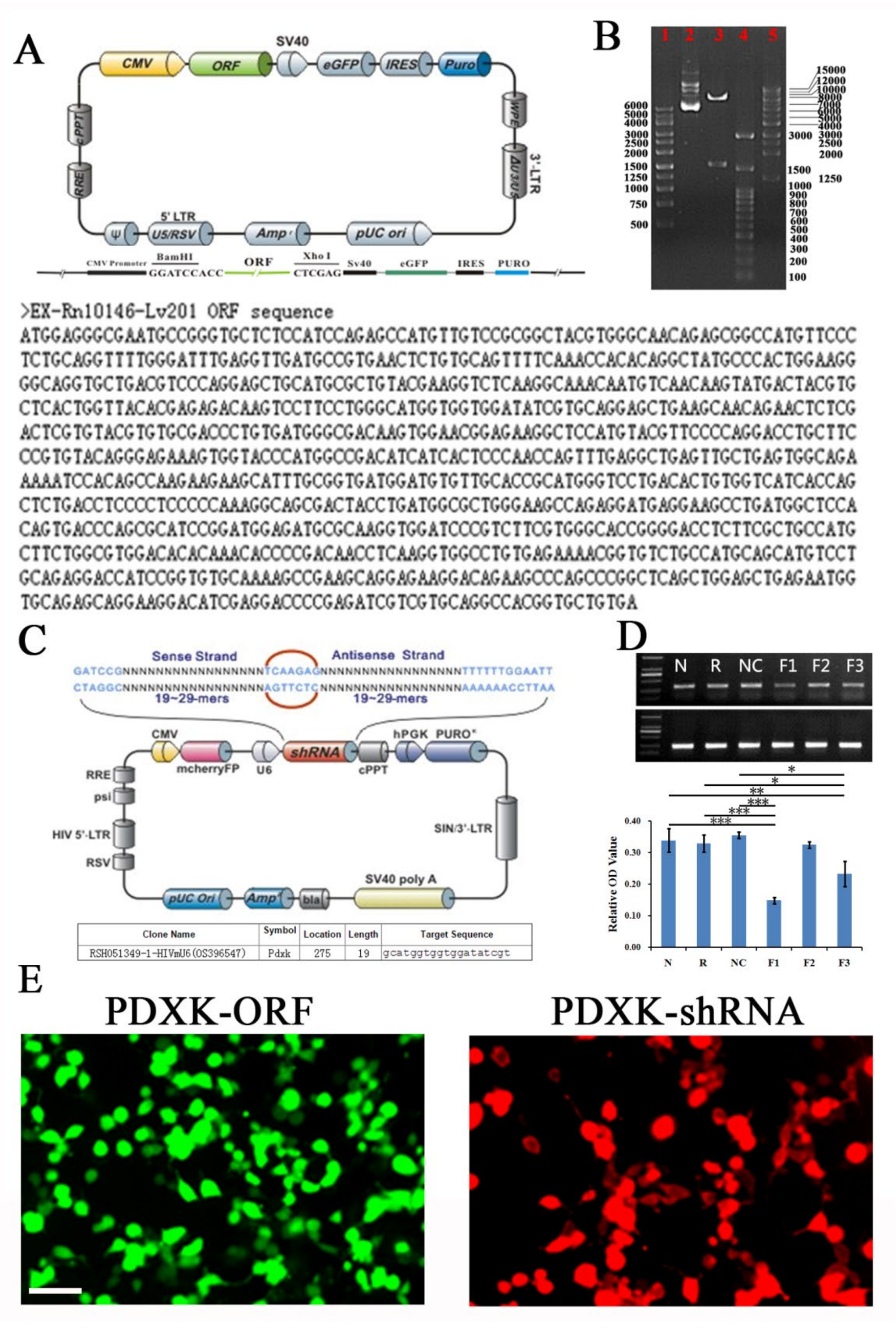

Supplementary Figure 1. PDXK-ORF/shRNA Lentivirus construction and Packaging. (A) Lentivirus plasmid information of EX-Rn10146-Lv201, including ORF sequence and restriction enzyme sites. CMV is the promoter of SNCA and EGFP is reporter gene. Puromycin and ampicillin are stable selection marker. **(B)** EX-Rn10146-Lv201 plasmid was digested by EcoRI and XhoI

restriction enzyme and run on 1.0% agarose gel. Lane1: DNA Ladder 6000. Lane2: EX-Rn10146-Lv201 plasmids. Lane3: EX-Rn10146-Lv201 plasmids were digested by EcoRI and XhoI. There are two expected bands (~1578/7963bp). Lane4: DNA ladder 3000. Lane5: DNA ladder 15000.**C** Lentivirus plasmid information of RSH051349-HIVmU6 and its interference sequence. CMV is the promoter and mcherryFP is reporter gene. Puromycin and ampicillin are stable selection marker. **(D)** The interference effectiveness of PDXK-siRNA was verified by RT-PCR in PC12 cells. N normal cells, R add transfection reagent into culture medium, NC random sequence added into culture medium, F1-3, add transfection reagent and one of three PDXK-siRNA fragment. The expression of PDXK was analyzed by Image J software and quantified, which suggested that F1 PDXK-siRNA was the most effective. Data were presented as mean ±SEM. *p<0.05 vs. normal. **(E)** EGFP (PDXK-ORF) and mcherryFP (PDXK-shRNA) are visualized under fluorescent microscope indicating successful packging in 293Tα cells. Scale bar= 50μm.

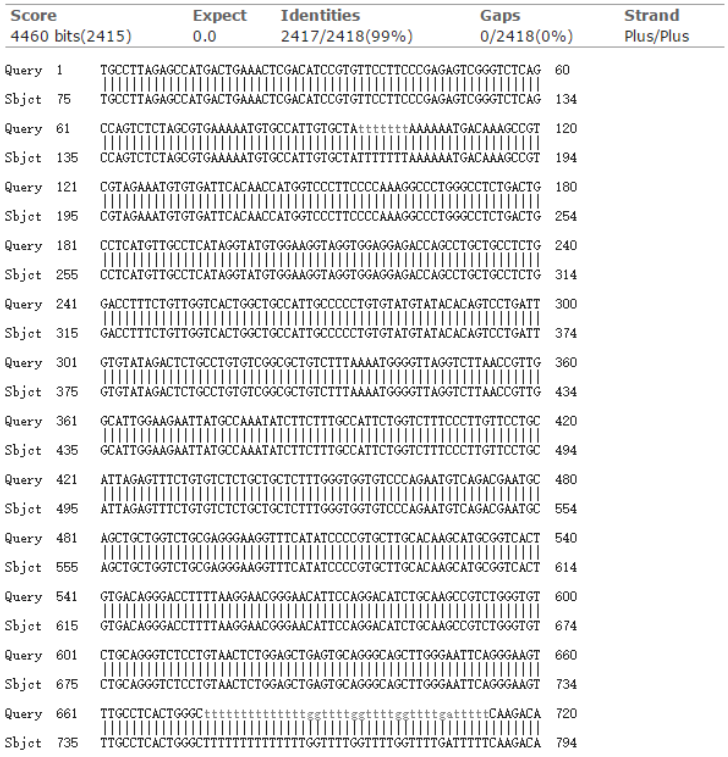

Supplementary Figure 2. The matching rate of PDXK 3 'UTR sequence and the sequencing results was 99% by using blast software, which indicated the recombination of hRluc-PDXK 3 'UTR

plasmid is successful. These experiments and data analysis were performed by Shanghai Biotechnology Co., Ltd. (Shanghai, China).

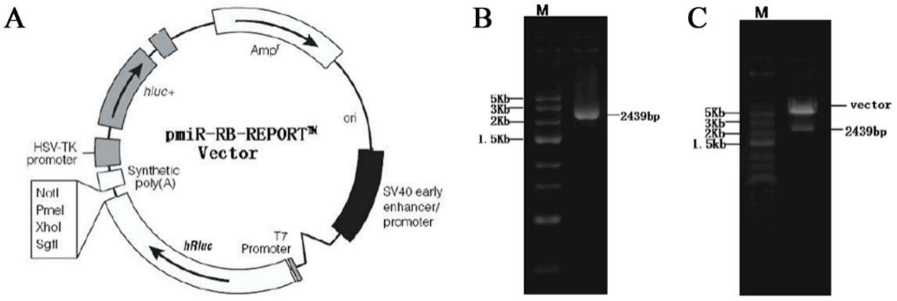

Supplementary Figure 3. Verification of PDXK 3 'UTR mutant vector. (A) The vector of PDXK 3 'UTR mutant. **(B)** The electrophoresis of PDXK. **M:** marker 5000. **(C)** Validation of plasmid enzyme digestion. M: marker 5000

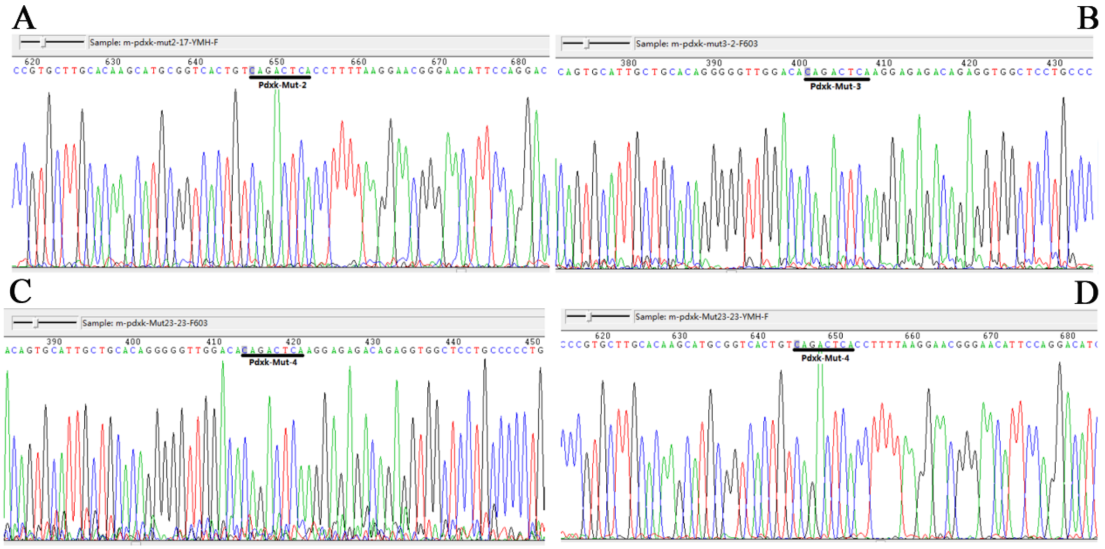

Supplementary Figure 4. The sequencing mapping of PDXK 3 'UTR mutant. (A) The sequencing mapping of PDXK 3 'UTR -Mut-2. **(B)** The sequencing mapping of PDXK 3 'UTR -Mut-3. **(C, D)** The sequencing mapping of PDXK3 'UTR -Mut-4

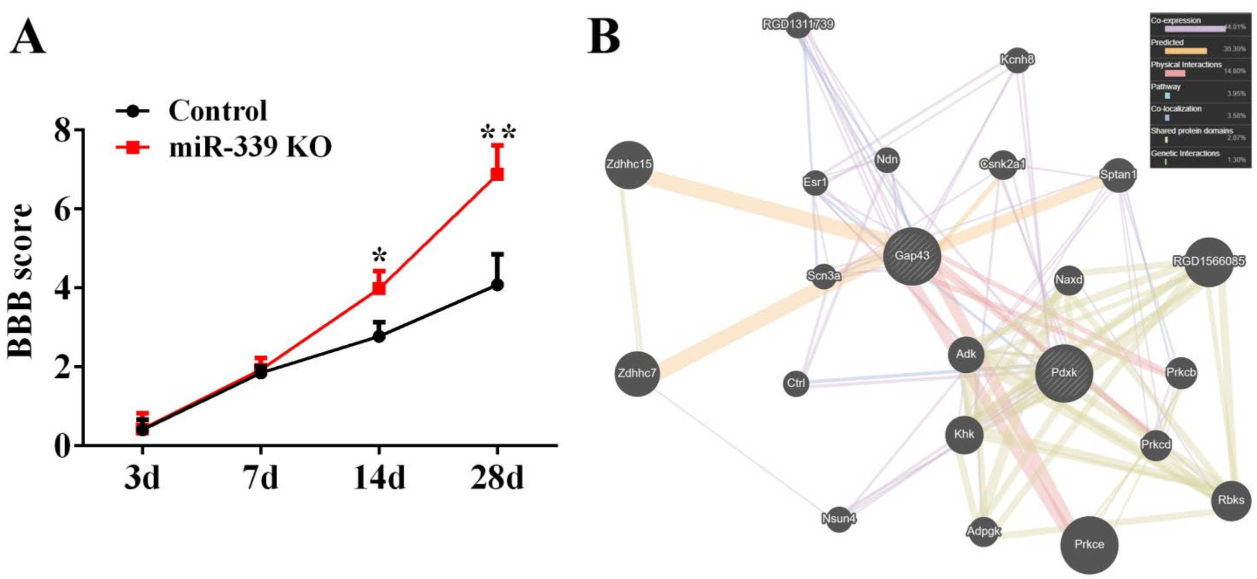

Supplementary Figure 5. The role of miR-339 knock-out in the functional recovery after SCT in rats and bioinformatics prediction with GAP43. (A) The locomotor function was assessed by BBB score between control group and miR-339 knock out group at 3 d, 7 d, 14 d and 28 d after SCT. Data were exhibited as mean ± SD. *P < 0.05, **P < 0.01. (B) PDXK is associated with GAP43 in SCT rats. The potential relation between PDXK and GAP43 was predicted by GeneMANIA (http://genemania.org/). Bioinformatics prediction revealed that there is a co-expression and direct relation between PDXK and GAP43. BBB=Basso, Beattie, and Bresnehan. KO = knock out. PDXK = Pyridoxal kinase. GAP43 = Growth associated protein-43. d = Day.
