# Supplementary material for: Corrigendum: MicroRNA339 Targeting PDXK Improves Motor Dysfunction and Promotes Neurite Growth in the Remote Cortex Subjected to Spinal Cord Transection
Source: Front Cell Dev Biol. 2022 Apr 13;10:877291. doi: 10.3389/fcell.2022.877291 (PMC9044487; doi:10.3389/fcell.2022.877291)
Supplement: Supplementary file 1 [file DataSheet2.PDF]

Vector Summary

**Vector ID**	VB161117-1054nkt
**Vector Name (official)**	pRP[CRISPR]-hCas9-U6>20nt_GCTACTGCATCGACAGCCGG
**Date Created (Pacific Time)**	2016-11-16
**Size**	8508 bp
**Vector Type**	Regular plasmid CRISPR vector (single gRNA)
**Inserted gRNA**	20nt_GCTACTGCATCGACAGCCGG
**Inserted Nuclease**	hCas9
**Target Sequence**	GCTACTGCATCGACAGCCGG
**Copy Number**	High
**Bacterial Resistance**	Ampicillin
**Cloning Host**	Stbl3

User Annotation of Vector

**Vector alias (from user)**	*None*
**Comment (from user)**	*None*

Vector Map

User-inserted region
Eukaryotic region
Bacterial region

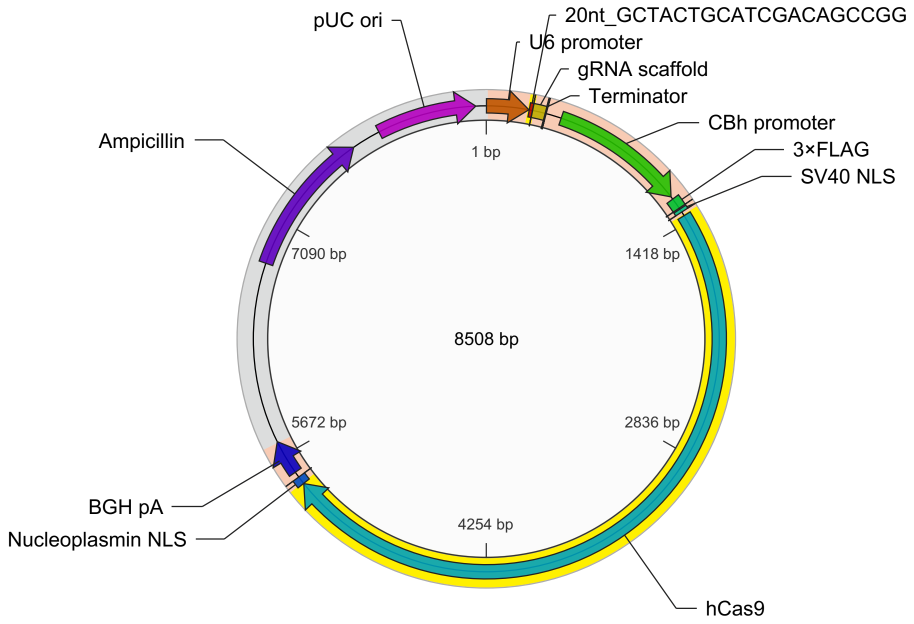

Vector Components

Component Name	Nucleotide Position	Full Name	Description
U6 promoter	**1-249**	Human U6 promoter	Allows high-level expression of gRNA.
20nt_GCTACTGCATCGACAGCCGG	251-270	20nt_GCTACTGCATCGACAGCCGG	Component entered by user
gRNA scaffold	**271-346**	Chimeric gRNA scaffold	Helps hCas9 bind to target DNA.
Terminator	347-352	U6 terminator	Allows transcription termination of gRNA.
CBh promoter	**443-1240**	Chicken betaActin hybrid promoter	Drives expression of hCas9.
3×FLAG	1256-1321	3 tandem flag epitopes	Allows to detect recombinant fusion proteins.
SV40 NLS	**1328-1348**	SV40 nuclear localization signal	Allows transportation of protein into the nucleus.
hCas9	1373-5473	Human codon-optimized Cas9	An RNA-guided DNA endonuclease enzyme associated with the CRISPR adaptive immunity system from Streptococcus pyogenes.
Nucleoplasmin NLS	**5474-5521**	Nucleoplasmin nuclear localization signal	Allows transportation of protein into the nucleus.
BGH pA	5555-5762	Bovine growth hormone polyadenylation	Allows transcription termination and polyadenylation of mRNA.
Ampicillin	**6828-7688**	Ampicillin resistance gene	Allows selection of the plasmid in E.coli.
pUC ori	7859-8447	pUC origin of replication	Permits high-copy replication and maintenance in E.coli.

Note: (c) denotes complementary strand.

User Annotation of Vector Components

Component Name	Comment by User
20nt_GCTACTGCATCGACAGCCGG	*None*
hCas9	*None*

Vector Sequence

1 GAGGGCCTAT TTCCCATGAT TCCTTCATAT TTGCATATAC GATACAAGGC TGTTAGAGAG
61 ATAATTGGAA TTAATTTGAC TGTAAACACA AAGATATTAG TACAAAATAC GTGACGTAGA
121 AAGTAATAAT TTCTTGGGTA GTTTGCAGTT TTAAAATTAT GTTTTAAAAT GGACTATCAT
181 ATGCTTACCG TAACTTGAAA GTATTTCGAT TTCTTGGCTT TATATATCTT GTGGAAAGGA
241 CGAAACACCG GCTACTGCAT CGACAGCCGG GTTTTAGAGC TAGAAATAGC AAGTTAAAAT
301 AAGGCTAGTC CGTTATCAAC TTGAAAAAGT GGCACCGAGT CGGTGCTTTT TTGTTTTAGA
361 GCTAGAAATA GCAAGTTAAA ATAAGGCTAG TCCGTTTTTA GCGCGTGCGC CAATTCTGCA
421 GACAAATGGC TCTAGAGGTA CCCGTTACAT AACTTACGGT AAATGGCCCG CCTGGCTGAC
481 CGCCCAACGA CCCCCGCCCA TTGACGTCAA TAGTAACGCC AATAGGGACT TTCCATTGAC
541 GTCAATGGGT GGAGTATTTA CGGTAAACTG CCCACTTGGC AGTACATCAA GTGTATCATA
601 TGCCAAGTAC GCCCCCTATT GACGTCAATG ACGGTAAATG GCCCGCCTGG CATTGTGCCC
661 AGTACATGAC CTTATGGGAC TTTCCTACTT GGCAGTACAT CTACGTATTA GTCATCGCTA
721 TTACCATGGT CGAGGTGAGC CCCACGTTCT GCTTCACTCT CCCCATCTCC CCCCCCTCCC
781 CACCCCCAAT TTTGTATTTA TTTATTTTTT AATTATTTTG TGCAGCGATG GGGGCGGGGG
841 GGGGGGGGGG GCGCGCGCCA GGCGGGGCGG GGCGGGGCGA GGGGCGGGGC GGGGCGAGGC
901 GGAGAGGTGC GGCGGCAGCC AATCAGAGCG GCGCGCTCCG AAAGTTTCCT TTTATGGCGA
961 GGCGGCGGCG GCGGCGGCCC TATAAAAAGC GAAGCGCGCG GCGGGCGGGA GTCGCTGCGC
1021 GCTGCCTTCG CCCCGTGCCC CGCTCCGCCG CCGCCTCGCG CCGCCCGCCC CGGCTCTGAC
1081 TGACCGCGTT ACTCCCACAG GTGAGCGGGC GGGACGGCCC TTCTCCTCCG GGCTGTAATT
1141 AGCTGAGCAA GAGGTAAGGG TTTAAGGGAT GGTTGGTTGG TGGGGTATTA ATGTTTAATT
1201 ACCTGGAGCA CCTGCCTGAA ATCACTTTTT TTCAGGTTGG ACCGGTGCCA CCATGGACTA
1261 TAAGGACCAC GACGGAGACT ACAAGGATCA TGATATTGAT TACAAAGACG ATGACGATAA
1321 GATGGCCCCA AAGAAGAAGC GGAAGGTCGG TATCCACGGA GTCCCAGCAG CCGACAAGAA
1381 GTACAGCATC GGCCTGGACA TCGGCACCAA CTCTGTGGGC TGGGCCGTGA TCACCGACGA
1441 GTACAAGGTG CCCAGCAAGA AATTCAAGGT GCTGGGCAAC ACCGACCGGC ACAGCATCAA
1501 GAAGAACCTG ATCGGAGCCC TGCTGTTCGA CAGCGGCGAA ACAGCCGAGG CCACCCGGCT
1561 GAAGAGAACC GCCAGAAGAA GATACACCAG ACGGAAGAAC CGGATCTGCT ATCTGCAAGA
1621 GATCTTCAGC AACGAGATGG CCAAGGTGGA CGACAGCTTC TTCCACAGAC TGGAAGAGTC
1681 CTTCCTGGTG GAAGAGGATA AGAAGCACGA GCGGCACCCC ATCTTCGGCA ACATCGTGGA
1741 CGAGGTGGCC TACCACGAGA AGTACCCCAC CATCTACCAC CTGAGAAAGA AACTGGTGGA
1801 CAGCACCGAC AAGGCCGACC TGCGGCTGAT CTATCTGGCC CTGGCCCACA TGATCAAGTT
1861 CCGGGGCCAC TTCCTGATCG AGGGCGACCT GAACCCCGAC AACAGCGACG TGGACAAGCT
1921 GTTCATCCAG CTGGTGCAGA CCTACAACCA GCTGTTCGAG GAAAACCCCA TCAACGCCAG
1981 CGGCGTGGAC GCCAAGGCCA TCCTGTCTGC CAGACTGAGC AAGAGCAGAC GGCTGGAAAA
2041 TCTGATCGCC CAGCTGCCCG GCGAGAAGAA GAATGGCCTG TTCGGAAACC TGATTGCCCT
2101 GAGCCTGGGC CTGACCCCCA ACTTCAAGAG CAACTTCGAC CTGGCCGAGG ATGCCAAACT
2161 GCAGCTGAGC AAGGACACCT ACGACGACGA CCTGGACAAC CTGCTGGCCC AGATCGGCGA
2221 CCAGTACGCC GACCTGTTTC TGGCCGCCAA GAACCTGTCC GACGCCATCC TGCTGAGCGA
2281 CATCCTGAGA GTGAACACCG AGATCACCAA GGCCCCCCTG AGCGCCTCTA TGATCAAGAG
2341 ATACGACGAG CACCACCAGG ACCTGACCCT GCTGAAAGCT CTCGTGCGGC AGCAGCTGCC
2401 TGAGAAGTAC AAAGAGATTT TCTTCGACCA GAGCAAGAAC GGCTACGCCG GCTACATTGA
2461 CGGCGGAGCC AGCCAGGAAG AGTTCTACAA GTTCATCAAG CCCATCCTGG AAAAGATGGA
2521 CGGCACCGAG GAACTGCTCG TGAAGCTGAA CAGAGAGGAC CTGCTGCGGA AGCAGCGGAC
2581 CTTCGACAAC GGCAGCATCC CCCACCAGAT CCACCTGGGA GAGCTGCACG CCATTCTGCG
2641 GCGGCAGGAA GATTTTTACC CATTCCTGAA GGACAACCGG GAAAAGATCG AGAAGATCCT
2701 GACCTTCCGC ATCCCCTACT ACGTGGGCCC TCTGGCCAGG GGAAACAGCA GATTCGCCTG
2761 GATGACCAGA AAGAGCGAGG AAACCATCAC CCCCTGGAAC TTCGAGGAAG TGGTGGACAA
2821 GGGCGCTTCC GCCCAGAGCT TCATCGAGCG GATGACCAAC TTCGATAAGA ACCTGCCCAA
2881 CGAGAAGGTG CTGCCCAAGC ACAGCCTGCT GTACGAGTAC TTCACCGTGT ATAACGAGCT
2941 GACCAAAGTG AAATACGTGA CCGAGGGAAT GAGAAAGCCC GCCTTCCTGA GCGGCGAGCA

3001 GAAAAAGGCC ATCGTGGACC TGCTGTTCAA GACCAACCGG AAAGTGACCG TGAAGCAGCT
3061 GAAAGAGGAC TACTTCAAGA AAATCGAGTG CTTCGACTCC GTGGAAATCT CCGGCGTGGA
3121 AGATCGGTTC AACGCCTCCC TGGGCACATA CCACGATCTG CTGAAAATTA TCAAGGACAA
3181 GGACTTCCTG GACAATGAGG AAAACGAGGA CATTCTGGAA GATATCGTGC TGACCCTGAC
3241 ACTGTTTGAG GACAGAGAGA TGATCGAGGA ACGGCTGAAA ACCTATGCCC ACCTGTTCGA
3301 CGACAAAGTG ATGAAGCAGC TGAAGCGGCG GAGATACACC GGCTGGGGCA GGCTGAGCCG
3361 GAAGCTGATC AACGGCATCC GGGACAAGCA GTCCGGCAAG ACAATCCTGG ATTTCCTGAA
3421 GTCCGACGGC TTCGCCAACA GAAACTTCAT GCAGCTGATC CACGACGACA GCCTGACCTT
3481 TAAAGAGGAC ATCCAGAAAG CCCAGGTGTC CGGCCAGGGC GATAGCCTGC ACGAGCACAT
3541 TGCCAATCTG GCCGGCAGCC CCGCCATTAA GAAGGGCATC CTGCAGACAG TGAAGGTGGT
3601 GGACGAGCTC GTGAAAGTGA TGGGCCGGCA CAAGCCCGAG AACATCGTGA TCGAAATGGC
3661 CAGAGAGAAC CAGACCACCC AGAAGGGACA GAAGAACAGC CGCGAGAGAA TGAAGCGGAT
3721 CGAAGAGGGC ATCAAAGAGC TGGGCAGCCA GATCCTGAAA GAACACCCCG TGGAAAACAC
3781 CCAGCTGCAG AACGAGAAGC TGTACCTGTA CTACCTGCAG AATGGGCGGG ATATGTACGT
3841 GGACCAGGAA CTGGACATCA ACCGGCTGTC CGACTACGAT GTGGACCATA TCGTGCCTCA
3901 GAGCTTTCTG AAGGACGACT CCATCGACAA CAAGGTGCTG ACCAGAAGCG ACAAGAACCG
3961 GGGCAAGAGC GACAACGTGC CCTCCGAAGA GGTCGTGAAG AAGATGAAGA ACTACTGGCG
4021 GCAGCTGCTG AACGCCAAGC TGATTACCCA GAGAAAGTTC GACAATCTGA CCAAGGCCGA
4081 GAGAGGCGGC CTGAGCGAAC TGGATAAGGC CGGCTTCATC AAGAGACAGC TGGTGGAAAC
4141 CCGGCAGATC ACAAAGCACG TGGCACAGAT CCTGGACTCC CGGATGAACA CTAAGTACGA
4201 CGAGAATGAC AAGCTGATCC GGGAAGTGAA AGTGATCACC CTGAAGTCCA AGCTGGTGTC
4261 CGATTTCCGG AAGGATTTCC AGTTTTACAA AGTGCGCGAG ATCAACAACT ACCACCACGC
4321 CCACGACGCC TACCTGAACG CCGTCGTGGG AACCGCCCTG ATCAAAAAGT ACCCTAAGCT
4381 GGAAAGCGAG TTCGTGTACG GCGACTACAA GGTGTACGAC GTGCGGAAGA TGATCGCCAA
4441 GAGCGAGCAG GAAATCGGCA AGGCTACCGC CAAGTACTTC TTCTACAGCA ACATCATGAA
4501 CTTTTTCAAG ACCGAGATTA CCCTGGCCAA CGGCGAGATC CGGAAGCGGC CTCTGATCGA
4561 GACAAACGGC GAAACCGGGG AGATCGTGTG GGATAAGGGC CGGGATTTTG CCACCGTGCG
4621 GAAAGTGCTG AGCATGCCCC AAGTGAATAT CGTGAAAAAG ACCGAGGTGC AGACAGGCGG
4681 CTTCAGCAAA GAGTCTATCC TGCCCAAGAG GAACAGCGAT AAGCTGATCG CCAGAAAGAA
4741 GGACTGGGAC CCTAAGAAGT ACGGCGGCTT CGACAGCCCC ACCGTGGCCT ATTCTGTGCT
4801 GGTGGTGGCC AAAGTGGAAA AGGGCAAGTC CAAGAAACTG AAGAGTGTGA AAGAGCTGCT
4861 GGGGATCACC ATCATGGAAA GAAGCAGCTT CGAGAAGAAT CCCATCGACT TTCTGGAAGC
4921 CAAGGGCTAC AAAGAAGTGA AAAAGGACCT GATCATCAAG CTGCCTAAGT ACTCCCTGTT
4981 CGAGCTGGAA AACGGCCGGA AGAGAATGCT GGCCTCTGCC GGCGAACTGC AGAAGGGAAA
5041 CGAACTGGCC CTGCCCTCCA AATATGTGAA CTTCCTGTAC CTGGCCAGCC ACTATGAGAA
5101 GCTGAAGGGC TCCCCCGAGG ATAATGAGCA GAAACAGCTG TTTGTGGAAC AGCACAAGCA
5161 CTACCTGGAC GAGATCATCG AGCAGATCAG CGAGTTCTCC AAGAGAGTGA TCCTGGCCGA
5221 CGCTAATCTG GACAAAGTGC TGTCCGCCTA CAACAAGCAC CGGGATAAGC CCATCAGAGA
5281 GCAGGCCGAG AATATCATCC ACCTGTTTAC CCTGACCAAT CTGGGAGCCC CTGCCGCCTT
5341 CAAGTACTTT GACACCACCA TCGACCGGAA GAGGTACACC AGCACCAAAG AGGTGCTGGA
5401 CGCCACCCTG ATCCACCAGA GCATCACCGG CCTGTACGAG ACACGGATCG ACCTGTCTCA
5461 GCTGGGAGGC GAC**AAAAGGC** **CGGCGGCCAC** **GAAAAAGGCC** **GGCCAGGCAA** **AAAAGAAAAA**
5521 **G**TAAGAATTC CTAGAGCTCG CTGATCAGCC TCGACTGTGC CTTCTAGTTG CCAGCCATCT
5581 GTTGTTTGCC CCTCCCCCGT GCCTTCCTTG ACCCTGGAAG GTGCCACTCC CACTGTCCTT
5641 TCCTAATAAA ATGAGGAAAT TGCATCGCAT TGTCTGAGTA GGTGTCATTC TATTCTGGGG
5701 GGTGGGGTGG GGCAGGACAG CAAGGGGGAG GATTGGGAAG AGAATAGCAG GCATGCTGGG
5761 GAGCGGCCGC AGGAACCCCT AGTGATGGAG TTGGCCACTC CCTCTCTGCG CGCTCGCTCG
5821 CTCACTGAGG CCGGGCGACC AAAGGTCGCC CGACGCCCGG GCTTTGCCCG GGCGGCCTCA
5881 GTGAGCGAGC GAGCGCGCAG CTGCCTGCAG GGGCGCCTGA TGCGGTATTT TCTCCTTACG
5941 CATCTGTGCG GTATTTCACA CCGCATACGT CAAAGCAACC ATAGTACGCG CCCTGTAGCG
6001 GCGCATTAAG CGCGGCGGGT GTGGTGGTTA CGCGCAGCGT GACCGCTACA CTTGCCAGCG
6061 CCTTAGCGCC CGCTCCTTTC GCTTTCTTCC CTTCCTTTCT CGCCACGTTC GCCGGCTTTC

6121 CCCGTCAAGC TCTAAATCGG GGGCTCCCTT TAGGGTTCCG ATTTAGTGCT TTACGGCACC
6181 TCGACCCCAA AAAACTTGAT TTGGGTGATG GTTCACGTAG TGGGCCATCG CCCTGATAGA
6241 CGGTTTTTCG CCCTTTGACG TTGGAGTCCA CGTTCTTTAA TAGTGGACTC TTGTTCCAAA
6301 CTGGAACAAC ACTCAACTCT ATCTCGGGCT ATTCTTTTGA TTTATAAGGG ATTTTGCCGA
6361 TTTCGGTCTA TTGGTTAAAA AATGAGCTGA TTTAACAAAA ATTTAACGCG AATTTTAACA
6421 AAATATTAAC GTTTACAATT TTATGGTGCA CTCTCAGTAC AATCTGCTCT GATGCCGCAT
6481 AGTTAAGCCA GCCCCGACAC CCGCCAACAC CCGCTGACGC GCCCTGACGG GCTTGTCTGC
6541 TCCCGGCATC CGCTTACAGA CAAGCTGTGA CCGTCTCCGG GAGCTGCATG TGTCAGAGGT
6601 TTTCACCGTC ATCACCGAAA CGCGCGAGAC GAAAGGGCCT CGTGATACGC CTATTTTTAT
6661 AGGTTAATGT CATGATAATA ATGGTTTCTT AGACGTCAGG TGGCACTTTT CGGGGAAATG
6721 TGCGCGGAAC CCCTATTTGT TTATTTTTCT AAATACATTC AAATATGTAT CCGCTCATGA
6781 GACAATAACC CTGATAAATG CTTCAATAAT ATTGAAAAAG GAAGAGT**ATG AGTATTCAAC**
6841 **ATTTCCGTGT CGCCCTTATT CCCTTTTTTG CGGCATTTTG CCTTCCTGTT TTTGCTCACC**
6901 **CAGAAACGCT GGTGAAAGTA AAAGATGCTG AAGATCAGTT GGGTGCACGA GTGGGTTACA**
6961 **TCGAACTGGA TCTCAACAGC GGTAAGATCC TTGAGAGTTT TCGCCCCGAA GAACGTTTTC**
7021 **CAATGATGAG CACTTTTAAA GTTCTGCTAT GTGGCGCGGT ATTATCCCGT ATTGACGCCG**
7081 **GGCAAGAGCA ACTCGGTCGC CGCATACACT ATTCTCAGAA TGACTTGGTT GAGTACTCAC**
7141 **CAGTCACAGA AAAGCATCTT ACGGATGGCA TGACAGTAAG AGAATTATGC AGTGCTGCCA**
7201 **TAACCATGAG TGATAACACT GCGGCCAACT TACTTCTGAC AACGATCGGA GGACCGAAGG**
7261 **AGCTAACCGC TTTTTTGCAC AACATGGGGG ATCATGTAAC TCGCCTTGAT CGTTGGGAAC**
7321 **CGGAGCTGAA TGAAGCCATA CCAAACGACG AGCGTGACAC CACGATGCCT GTAGCAATGG**
7381 **CAACAACGTT GCGCAAACTA TTAACTGGCG AACTACTTAC TCTAGCTTCC CGGCAACAAT**
7441 **TAATAGACTG GATGGAGGCG GATAAAGTTG CAGGACCACT TCTGCGCTCG GCCCTTCCGG**
7501 **CTGGCTGGTT TATTGCTGAT AAATCTGGAG CCGGTGAGCG TGGAAGCCGC GGTATCATTG**
7561 **CAGCACTGGG GCCAGATGGT AAGCCCTCCC GTATCGTAGT TATCTACACG ACGGGGAGTC**
7621 **AGGCAACTAT GGATGAACGA AATAGACAGA TCGCTGAGAT AGGTGCCTCA CTGATTAAGC**
7681 **ATTGGTAA**CT GTCAGACCAA GTTTACTCAT ATATACTTTA GATTGATTTA AAACTTCATT
7741 TTTAATTTAA AAGGATCTAG GTGAAGATCC TTTTTGATAA TCTCATGACC AAAATCCCTT
7801 AACGTGAGTT TTCGTTCCAC TGAGCGTCAG ACCCCGTAGA AAAGATCAAA GGATCTTCTT
7861 GAGATCCTTT TTTTCTGCGC GTAATCTGCT GCTTGCAAAC AAAAAAACCA CCGCTACCAG
7921 CGGTGGTTTG TTTGCCGGAT CAAGAGCTAC CAACTCTTTT TCCGAAGGTA ACTGGCTTCA
7981 GCAGAGCGCA GATACCAAAT ACTGTTCTTC TAGTGTAGCC GTAGTTAGGC CACCACTTCA
8041 AGAACTCTGT AGCACCGCCT ACATACCTCG CTCTGCTAAT CCTGTTACCA GTGGCTGCTG
8101 CCAGTGGCGA TAAGTCGTGT CTTACCGGGT TGGACTCAAG ACGATAGTTA CCGGATAAGG
8161 CGCAGCGGTC GGGCTGAACG GGGGGTTCGT GCACACAGCC CAGCTTGGAG CGAACGACCT
8221 ACACCGAACT GAGATACCTA CAGCGTGAGC TATGAGAAAG CGCCACGCTT CCCGAAGGGA
8281 GAAAGGCGGA CAGGTATCCG GTAAGCGGCA GGGTCGGAAC AGGAGAGCGC ACGAGGGAGC
8341 TTCCAGGGGG AAACGCCTGG TATCTTTATA GTCCTGTCGG GTTTCGCCAC CTCTGACTTG
8401 AGCGTCGATT TTTGTGATGC TCGTCAGGGG GGCGGAGCCT ATGGAAAAAC GCCAGCAACG
8461 CGGCCTTTTT ACGGTTCCTG GCCTTTTGCT GGCCTTTTGC TCACATGT

Qualification by Sequencing

Primer Name	Primer Sequence	Strand
gRNA-F1	CGGAGCCTATGGAAAAACGC	Forward
hCas9-R1	CAGATCCGGTTCTTCCGTCT	Reverse
